# Inositol polyphosphates regulate and predict yeast pseudohyphal growth phenotypes

**DOI:** 10.1371/journal.pgen.1007493

**Published:** 2018-06-25

**Authors:** Kaitlyn L. Norman, Christian A. Shively, Amberlene J. De La Rocha, Nebibe Mutlu, Sukanya Basu, Paul J. Cullen, Anuj Kumar

**Affiliations:** 1 Department of Molecular, Cellular, and Developmental Biology, University of Michigan, Ann Arbor, Michigan, United States of America; 2 Program in Cellular and Molecular Biology, University of Michigan, Ann Arbor, Michigan, United States of America; 3 Department of Biological Sciences, University at Buffalo, Buffalo, New York, United States of America; Oregon State University, UNITED STATES

## Abstract

Pseudohyphal growth is a nutrient-regulated program in which budding yeast form multicellular filaments of elongated and connected cells. Filamentous growth is required for virulence in pathogenic fungi and provides an informative model of stress-responsive signaling. The genetics and regulatory networks modulating pseudohyphal growth have been studied extensively, but little is known regarding the changes in metabolites that enable pseudohyphal filament formation. Inositol signaling molecules are an important class of metabolite messengers encompassing highly phosphorylated and diffusible inositol polyphosphates (InsPs). We report here that the InsP biosynthesis pathway is required for wild-type pseudohyphal growth. Under nitrogen-limiting conditions that can induce filamentation, InsPs exhibit characteristic profiles, distinguishing the InsP_7_ pyrophosphate isoforms 1PP-InsP_5_ and 5PP-InsP_5_. Deletion and overexpression analyses of InsP kinases identify elevated levels of 5PP-InsP_5_ relative to 1PP-InsP_5_ in mutants exhibiting hyper-filamentous growth. Overexpression of *KCS1*, which promotes formation of inositol pyrophosphates, is sufficient to drive pseudohyphal filamentation on medium with normal nitrogen levels. We find that the kinases Snf1p (AMPK), Kss1p, and Fus3p (MAPKs), required for wild-type pseudohyphal growth, are also required for wild-type InsP levels. Deletion analyses of the corresponding kinase genes indicate elevated InsP_3_ levels and an absence of exaggerated 5PP-InsP_5_ peaks in trace profiles from *snf1*Δ/Δ and *kss1*Δ/Δ mutants exhibiting decreased pseudohyphal filamentation. Elevated 5PP-InsP_5_:1PP-InsP_5_ ratios are present in the hyperfilamentous *fus3* deletion mutant. Collectively, the data identify the presence of elevated 5PP-InsP_5_ levels relative to other inositol pyrophosphates as an *in vivo* marker of hyper-filamentous growth, while providing initial evidence for the regulation of InsP signaling by pseudohyphal growth kinases.

## Author summary

Changes in metabolite levels underlie important biological processes, including cellular responses to nutrient stress. One such response encompasses the nitrogen stress-induced transition of budding yeast cells into multicellular filaments, relevant as a model of directional growth and fungal pathogenesis. We report here that a conserved family of charged lipid-derived metabolites, inositol polyphosphates, exhibits characteristic changes as yeast cell form filaments in response to conditions of nitrogen limitation. The ratios of doubly charged inositol pyrophosphates consistently match with the degree of filament formation. Enzymes of the inositol polyphosphate synthesis pathway are required for filament formation, and inositol polyphosphate levels are dependent on kinases that enable wild-type filamentation. Our data indicate that inositol polyphosphates mark filamentous growth states, highlighting a new regulatory role for these ubiquitous eukaryotic second messengers.

## Introduction

The transition from unicellular yeast to growth in multicellular filaments is characteristic of many fungi [1–4]. In the budding yeast *Saccharomyces cerevisiae* nitrogen limitation or growth in an alternate carbon source induces cell elongation, unipolar budding, and altered cell-cell adhesion, resulting in the formation of pseudohyphal filaments that presumably benefits the organism as a scavenging mechanism [5–7]. Yeast pseudohyphal growth is relevant as a model of polarized growth, and in the related opportunistic human fungal pathogen *Candida albicans*, filamentous development is required for virulence [8, 9]. Classic studies have identified a core set of conserved signaling modules regulating yeast pseudohyphal growth, including the Kss1p mitogen-activated protein kinase (MAPK) cascade, the AMP-activated kinase family member Snf1p, and the Ras2p/cAMP-dependent protein kinase A (PKA) pathway [10–17]. Genomic screens using loss-of-function mutants and overexpression libraries have identified a broader set of genes required for pseudohyphal growth [18–20], but little is known regarding the changes in metabolite levels that underlie the filamentous growth transition.

**Fig 1:**
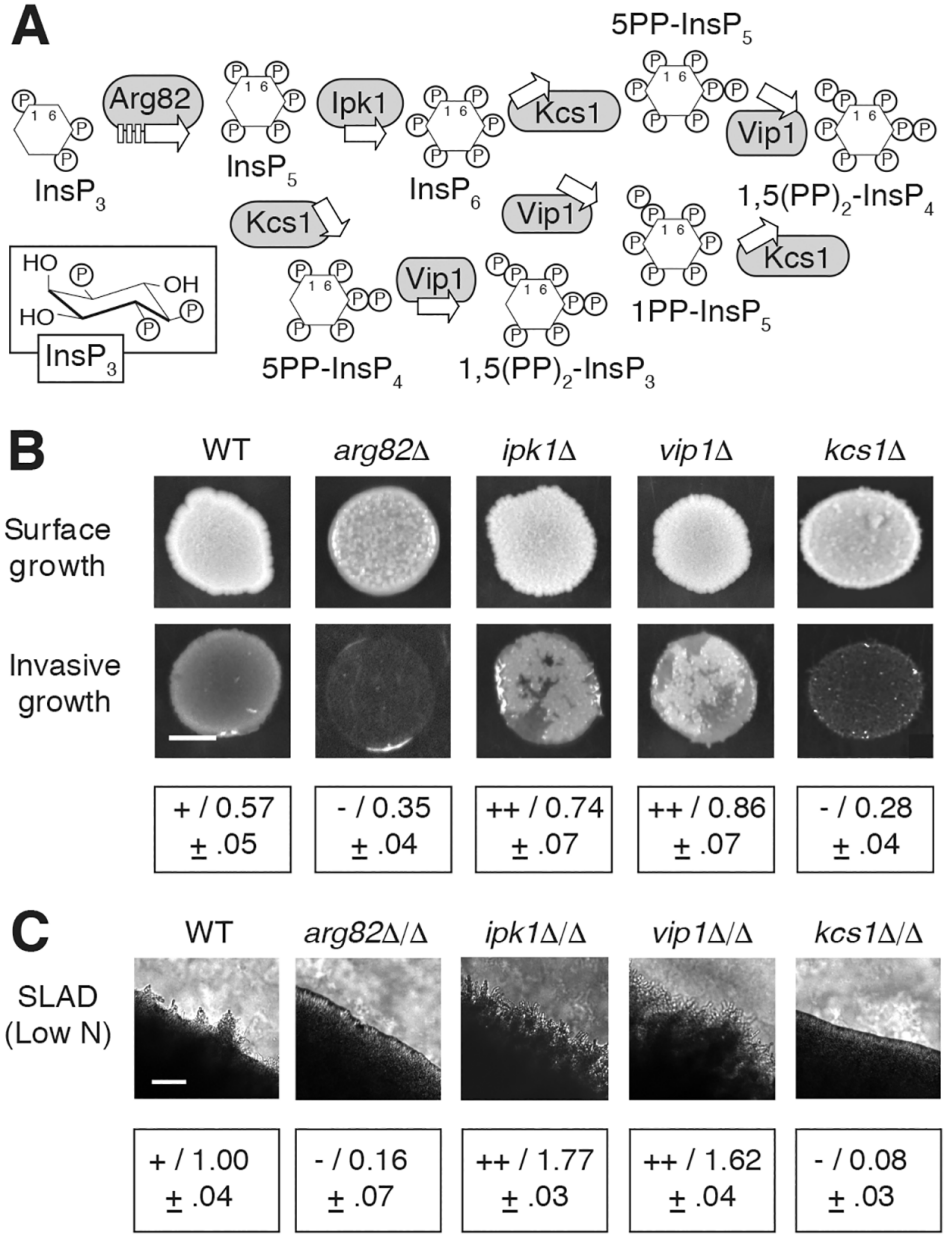
Genes encoding kinases in the InsP biosynthesis pathway are required for wild-type filamentous growth. **(A)** Biosynthetic pathway for InsPs and inositol pyrophosphates. The boat conformation for InsP_3_ is boxed; InsPs and inositol pyrophosphates are otherwise indicated as inositol hexagons with designated phosphates. InsP kinases acting in the pathway are boxed in gray. **(B)** Invasive growth phenotypes for haploid strains deleted of the indicated InsP kinase genes. Surface cells are washed from the spotted culture to identify invasive growth. Invasive growth was quantified as the pixel intensity of washed cultures relative to the pixel intensity of the spotted culture prior to washing. Measurements indicate the mean and standard deviation of three replicates; measurements for each replicate are provided in S5 Table. Hyper-invasive growth relative to wild type is indicated with a “++” (washed to pre-washed pixel intensity ratio of greater than 0.7). Wild-type pseudohyphal growth is indicated with a “+” (pixel intensity ratio between 0.5 and 0.7), and decreased invasive growth is indicated with a “-” (pixel intensity of washed spot relative to pre-washed spot less than 0.5). Scale bar, 2 mm. **(C)** Surface pseudohyphal filamentation is shown for the indicated homozygous diploid deletion strains grown on low-nitrogen SLAD medium. Pseudohyphal filaments are evident as irregularly shaped projections at the circumference of the imaged colony. Pseudohyphal filamentation was quantified as follows: the circumference of a defined area of the colony was measured and is presented as the ratio to the circumference of a corresponding area of a wild-type colony under pseudohyphal growth-inducing conditions of low nitrogen. Ratios indicate the mean of three replicates with standard deviation. Individual ratio measurements for each replicate are presented in S6 Table. Mean ratios were binned as follows: hyperfilamentous (++), ratios greater than 1.5; wild-type (+), ratios between 0.75 and 1.5; hypofilamentous (-), ratios less than 0.75. Scale bar, 500 μm.

Inositol polyphosphates (InsPs or IPs) are highly charged, lipid-derived metabolites that act as second messengers in organisms throughout the eukaryotic kingdom [21]. InsP biosynthesis is initiated by the enzyme phospholipase C, which cleaves the six-carbon cyclitol inositol from phosphatidyl-inositol 4,5-bisphosphate, yielding diffusible inositol 1,4,5-trisphosphate (InsP_3_) [22]. InsP_3_ has long been recognized to induce the early mobilization of calcium from the endoplasmic reticulum in many metazoans, although a similar role is not evident in budding yeast [23]. As indicated in Fig 1A, InsP_3_ is converted into inositol hexakisphosphate (InsP_6_) through a series of phosphorylation reactions catalyzed by the InsP kinases Arg82p and Ipk1p [24]. InsP_6_ is the most abundant InsP with levels ranging from 10–100 μM in most eukaryotes [25]. Pyrophosphorylated InsP molecules containing energy-rich diphosphate bonds are generated from InsP_5_ and InsP_6_ through the kinase activities of Kcs1p and Vip1p [26, 27]. Inositol pyrophosphate pools are turned over, such that up to 50% of human InsP_6_ is converted into pyrophosphate molecules per hour. With this turnover rate, inositol pyrophosphate levels are low, constituting 1–5% of InsP_6_ levels [28, 29]. Inositol pyrophosphate turnover is in part achieved through the action of three InsP phosphatases in yeast: Ddp1p, Siw14p, and Vip1p, which contains a putative phosphatase domain [30–32].

InsPs, and particularly inositol pyrophosphates, have been found to regulate many cellular processes, encompassing telomere maintenance, insulin signaling, cell migration, endocytosis, mRNA export, and chromatin modification [33–39]. Recent studies in yeast and *Arabidopsis* suggest that inositol pyrophosphates respond to cellular inorganic phosphate levels, acting as sensors for the regulation of phosphate homeostasis in eukaryotes [40]. Interestingly, inositol pyrophosphates can generate pyrophosphorylated proteins through the enzyme-independent transfer of their β-phosphoryl group onto phosphoprotein targets [41, 42]. These studies highlight the importance of inositol polyphosphates as metabolite messengers, but questions remain regarding the regulation of InsPs and the breadth of their role in cell signaling.

In previous work, we utilized quantitative phosphoproteomics to identify yeast proteins within kinase-based pseudohyphal growth signaling networks [43]. The data identified several InsP kinases, leading us to consider the possibility that inositol polyphosphate signaling may contribute to pseudohyphal growth, and more broadly, to the cellular response to nitrogen limitation. Our analyses here support this notion, indicating that InsP signaling is required for wild-type pseudohyphal growth and that the respective ratios of inositol pyrophosphates, in particular, change stereotypically in filamentous growth mutants, to the point of being predictive of exaggerated pseudohyphal growth states in a strain that is otherwise competent for filament formation. We further find that pseudohyphal growth kinases of the MAPK and AMPK families are required for wild-type levels of InsPs, suggesting a regulatory link between nutrient-responsive signaling and InsP metabolite messengers.

## Results

### InsP kinases are required for wild-type pseudohyphal growth

We previously used proteome-wide quantitative mass spectrometry to identify proteins differentially phosphorylated in yeast mutants carrying a catalytically defective allele of a kinase required for pseudohyphal growth [43]. Mutant alleles of eight kinase genes were analyzed in the study (*elm1*-K117R, *fus3*-K42R, *kss1*-K42R, *snf1*-K84R, *ste7*-K220R, *ste11*-K444R, *ste20*-K649R, and *tpk2*-K99R). The InsP kinase Arg82p and the inositol pyrophosphate kinases Kcs1p and Vip1p were differentially phosphorylated in the *kss1*-K42R and *snf1*-K84R mutants under conditions of reduced nitrogen availability (S1 Table). These results led us to further explore a possible role for InsP signaling in the yeast pseudohyphal growth response.

To assess the functional significance of InsPs in pseudohyphal growth, we generated haploid and homozygous diploid yeast deletion mutants in the filamentous Σ1278b genetic background for each gene encoding a kinase in the InsP biosynthetic pathway. Haploid deletion mutants were assayed for the ability to invade agar, and surface-spread filamentation was characterized in diploid mutants grown on low-nitrogen medium with a reduced concentration of ammonium sulfate (Fig 1B). Cell morphology of the diploid deletion mutants is indicated in S1 Fig. Invasive and surface-spread phenotypes were consistent for each mutant. Mutants deleted for *ARG82*, which lack InsPs and inositol pyrophosphates downstream of InsP_3_, exhibited decreased haploid invasive and diploid pseudohyphal development. This is consistent with the observation that diploid cells lacking *PLC1* do not form pseudohyphae on low-nitrogen media [44]. Strains deleted for *IPK1* and *VIP1* showed elevated pseudohyphal growth. Deletion of *KCS1* resulted in decreased invasion and surface pseudohyphal growth relative to wild type. Considered together, the deletion analyses indicate that the presence of each InsP kinase is required for wild-type pseudohyphal growth and that perturbation of InsP biosynthesis can result in either decreased or increased pseudohyphal filamentation, with the respective phenotype likely reflecting the accumulation of a given InsP species or set of InsPs. Subsequent data presented here are consistent with this conclusion.

### InsP profiles under pseudohyphal growth conditions are distinct and distinguish InsP_7_ isoforms

**Fig 2:**
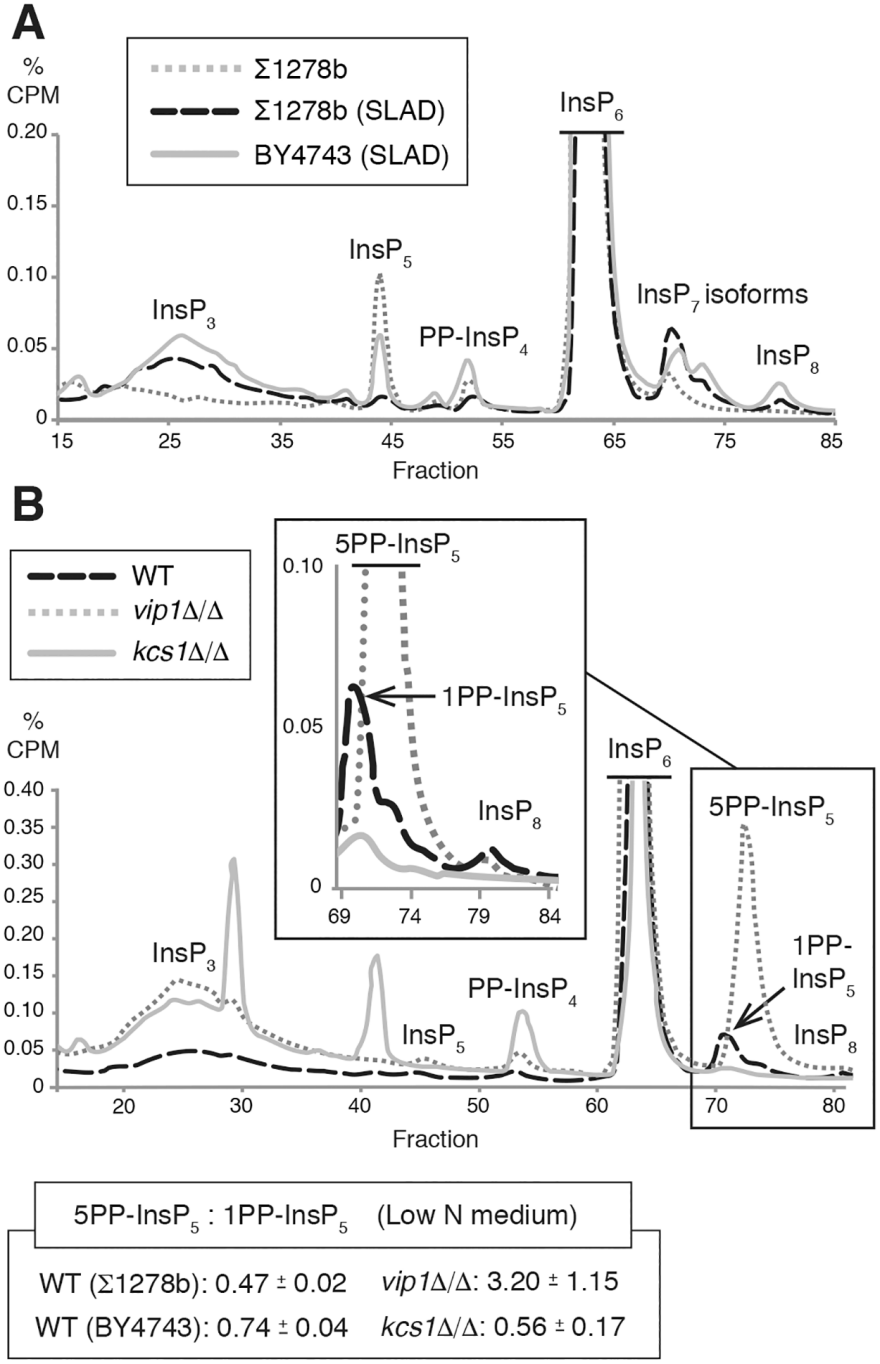
Analysis of InsP levels in yeast pseudohyphal growth. A representative profile is shown from at least two independent biological replicates for each strain and growth condition tested. **(A)** InsP profiles are altered under conditions that induce pseudohyphal filamentation (growth in low-nitrogen SLAD media for eight hours), with distinguishable InsP_7_ isoforms. InsP profiles are shown for a diploid wild-type filamentous strain grown under standard conditions (Σ1278b), a wild-type filamentous strain grown in low-nitrogen media for eight hours (Σ1278b SLAD), and a non-filamentous wild-type strain grown in low-nitrogen media for eight hours (BY4743 SLAD). **(B)** Representative InsP profiles of homozygous diploid *vip1*Δ/Δ and *kcs1*Δ/Δ mutants grown in low-nitrogen media compared to wild type. Profile regions corresponding to InsP_7_ and InsP_8_ are enlarged in the inset box. Each trace indicates counts per min (CPM) as a percentage of total CPM, with background subtracted. Elution fractions are indicated on the X-axis. Values used to generate the InsP traces are provided in S1 Dataset. The mean ratio of 5PP-IP_5_ to 1PP-IP_5_ is listed with standard deviation for each strain. Individual ratios of InsP_7_ isoforms for each tested replicate are provided in S7 Table.

To identify the relative levels of InsP species during pseudohyphal growth, we used the approach of Azvedo and Saiardi [45] in which yeast strains are cultured in media with radiolabeled *myo*-inositol; labeled inositol is taken up by yeast and subsequently metabolized into downstream InsPs and inositol pyrophosphates. InsP levels were profiled by this method in wild-type filamentous yeast (Σ1278b) grown in normal media and under pseudohyphal growth-inducing conditions in low-nitrogen media with reduced ammonium sulfate. Notable characteristics were evident in the profiles under conditions of low nitrogen (Fig 2A). In particular, two peaks corresponding to InsP_7_ isoforms were present, whereas only one InsP_7_ peak has classically been reported in strains grown in media with normal levels of ammonium sulfate. In addition, the peak corresponding to InsP_5_ was absent under low-nitrogen conditions, and an increased and broad trace corresponding to InsP_3_ was observed. This broad InsP_3_ trace presumably indicates InsP_3_ or InsP_4_ isoforms that we were not able to characterize with our standard deletion strains. To consider if the changes are specific to pseudohyphal growth or represent a more general response to nitrogen limitation, we determined the InsP profile for a non-filamentous strain (BY4743) on identical low-nitrogen growth media (Fig 2A). The InsP traces exhibited a number of similarities, but in the non-filamentous strain, InsP_5_ levels did not drop as substantially, and the peaks corresponding to PP-InsP_4_ and InsP_8_ were elevated relative to those observed from the filamentous strain on low-nitrogen media.

With two InsP_7_ isoforms detected in wild type under conditions of low nitrogen availability, we clarified the identity of each isoform peak by profiling InsP levels in homozygous diploid mutants deleted singly for the inositol pyrophosphate kinases *VIP1* and *KCS1* (Fig 2B). Deletion of *VIP1* under low-nitrogen conditions resulted in a strikingly large increase in the second InsP_7_ isoform peak, presumably corresponding to Kcs1p-produced 5PP-InsP_5_. Deletion of *KCS1* resulted in decreased InsP_7_ isoforms. The peak corresponding to the second InsP_7_ isoform that was elevated in *vip1*Δ/Δ was lost in traces of *kcs1*Δ/Δ; the peak corresponding to the first InsP_7_ isoform was decreased but still evident in the trace from *kcs1*Δ/Δ, and hence is most likely Vip1p-produced 1PP-InsP_5_. Analysis of *kcs1*Δ/Δ also identified elevated levels of PP-InsP_4_ and two profile peaks that were not observed in other InsP kinase pathway mutants that we could not conclusively identify from alignment with our standards. As indicated in Fig 1, the *vip1*Δ/Δ mutant that accumulates 5PP-InsP_5_ exhibited exaggerated pseudohyphal filamentation, while the *kcs1*Δ/Δ mutant that lacks an elevated ratio of 5PP-InsP_5_ to 1PP-InsP_5_ was hypofilamentous. Collectively, these data are consistent with the second InsP_7_ isoform peak corresponding to 5PP-InsP_5_ and suggest that the relative levels of pyrophosphorylated InsP_7_ isoforms correlate with pseudohyphal growth phenotypes.

### The kinase domain of Vip1p suppresses pseudohyphal growth

**Fig 3:**
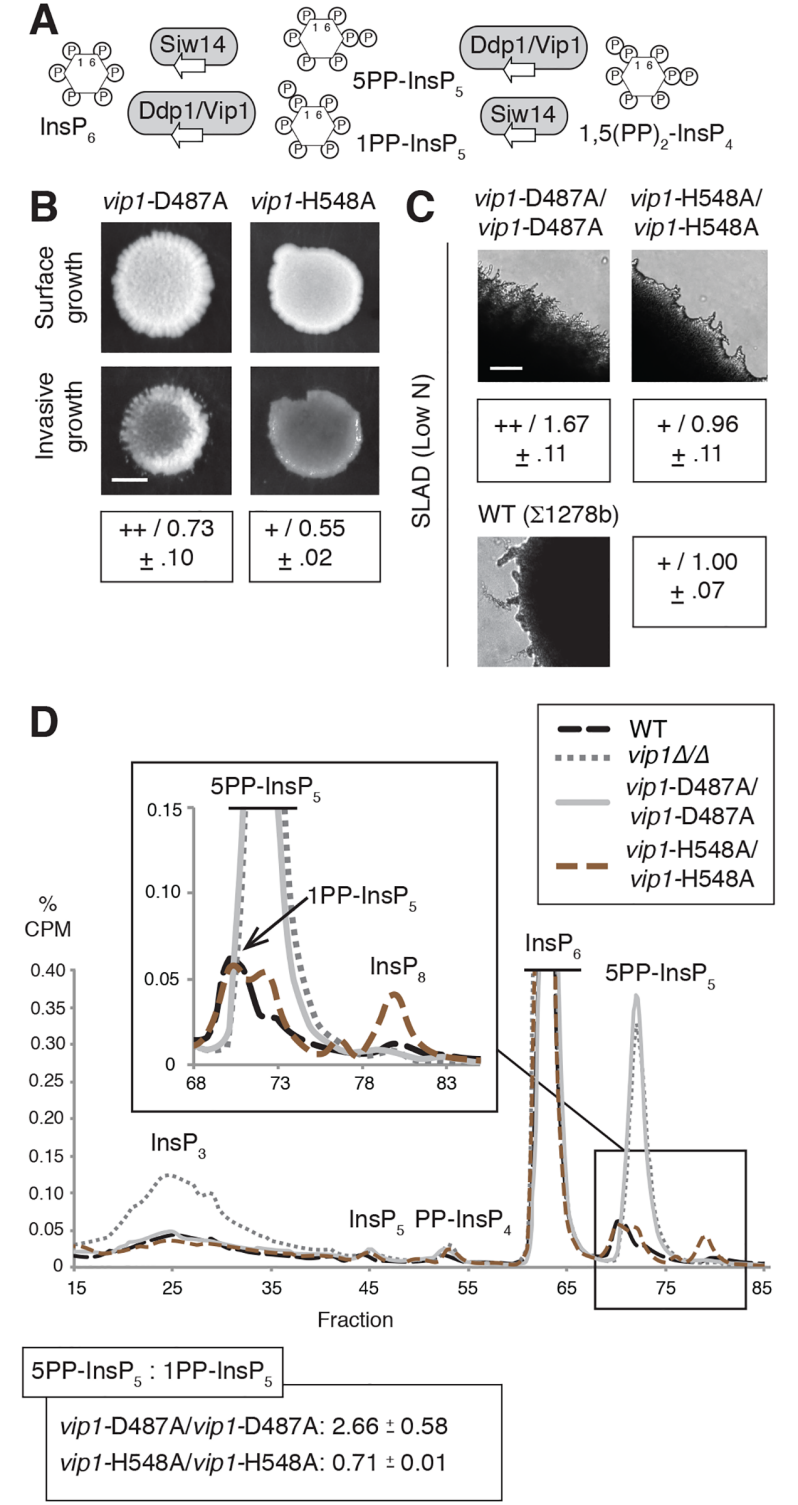
Mutation of the Vip1p kinase domain results in exaggerated pseudohyphal growth. **(A)** Diagram indicating phosphatases affecting inositol pyrophosphates. Vip1p phosphatase activity acts redundantly with Ddp1p. **(B)** Haploid invasive growth phenotypes are shown for strains containing a kinase-defective *vip1* allele (D487A) and phosphatase-defective *vip1* allele (H548A), respectively. For comparison, haploid wild type and *vip1*Δ mutants are included. Assays were performed and quantified as described previously. Mean values with standard deviation for three replicates are provided. Scale bar, 2 mm. **(C)** Surface pseudohyphal filamentation is shown for the indicated homozygous diploid strains grown on low-nitrogen SLAD medium. Mean values with standard deviation for three replicates are provided. Scale bar, 500 μm. **(D)** Representative InsP profiles of kinase-defective and phosphatase-defective *vip1* mutants grown in low-nitrogen minimal media for eight hours. Diploid wild type and *vip1*Δ/Δ strains grown under identical conditions are shown for comparison. Plots of InsP_7_ isoforms and InsP_8_ are enlarged in the boxed inset.

InsP levels are established through the actions of both kinases and phosphatases; phosphatases in the InsP biosynthesis pathway (Siw14p, Ddp1p, and Vip1p) are indicated in Fig 3A. Vip1p exhibits both InsP kinase and phosphatase activity. Vip1p contains an amino-terminal RimK/ATP-grasp domain responsible for phosphorylating the 1 position of InsP_6_ and a C-terminal phosphatase-like domain that acts to dephosphorylate molecules produced by Vip1p itself [31]. To determine if the elevated filamentation in *vip1*Δ/Δ resulted from loss of its kinase activity or phosphatase activity, we constructed chromosomal point mutations encoding kinase-defective (*vip1*-D487A) or phosphatase-defective (*vip1*-H548A) forms of Vip1p, in which the indicated conserved catalytically important residue was mutated to alanine. Haploid *vip1*-D487A and diploid *vip1*-D487A/D487A strains showed increased invasive growth and surface-spread filamentation, respectively (Fig 3B and 3C). Cell morphology images are presented in S1 Fig. The *vip1*-H548A strains exhibited wild-type filamentous growth. InsP profiles of *vip1*-D487A/D487A indicated a marked increase in 5PP-InsP_5_ levels relative to 1PP-InsP_5_ (Fig 3D). In contrast, the *vip1*-H548A/H548A mutant exhibited a 5PP-InsP_5_:1PP-InsP_5_ ratio of less than 1. Neither point mutant replicated the large increase in InsP_3_ levels detected in *vip1*Δ/Δ. From these results, we conclude that the kinase domain of Vip1p, but not its phosphatase-like domain, inhibits pseudohyphal growth, and that mutations impairing Vip1p kinase activity, but not its phosphatase activity, result in an elevated ratio of 5PP-InsP_5_ to 1PP-InsP_5_.

To consider the molecular basis of the pseudohyphal growth phenotypes in the *vip1* mutants, we assessed mRNA levels of the flocculence gene *FLO11*. The *FLO11* gene encodes a GPI-anchored cell surface flocculin, often used as a molecular marker of pseudohyphal growth. Deletion of *FLO11* yields a pseudohyphal growth defect under conditions of low nitrogen, and its complex promoter has been long studied as an important regulatory point in the pseudohyphal response [6]. Transcription of *FLO11* is controlled through MAPK signaling (positively through Kss1p and negatively through Fus3p), the PKA pathway, and the Snf1p signaling system. We observed a strong increase in *FLO11* mRNA levels in the hyperfilamentous *vip1*-D487A mutant relative to wild-type under conditions of low nitrogen, but less marked changes in *FLO11* mRNA levels in the phosphatase-defective *vip1*-H548A/H548A strain (S2 Table).

### Loss of the InsP phosphatase Siw14p results in elevated 5PP-InsP_5_ levels and hyper-filamentous growth

**Fig 4:**
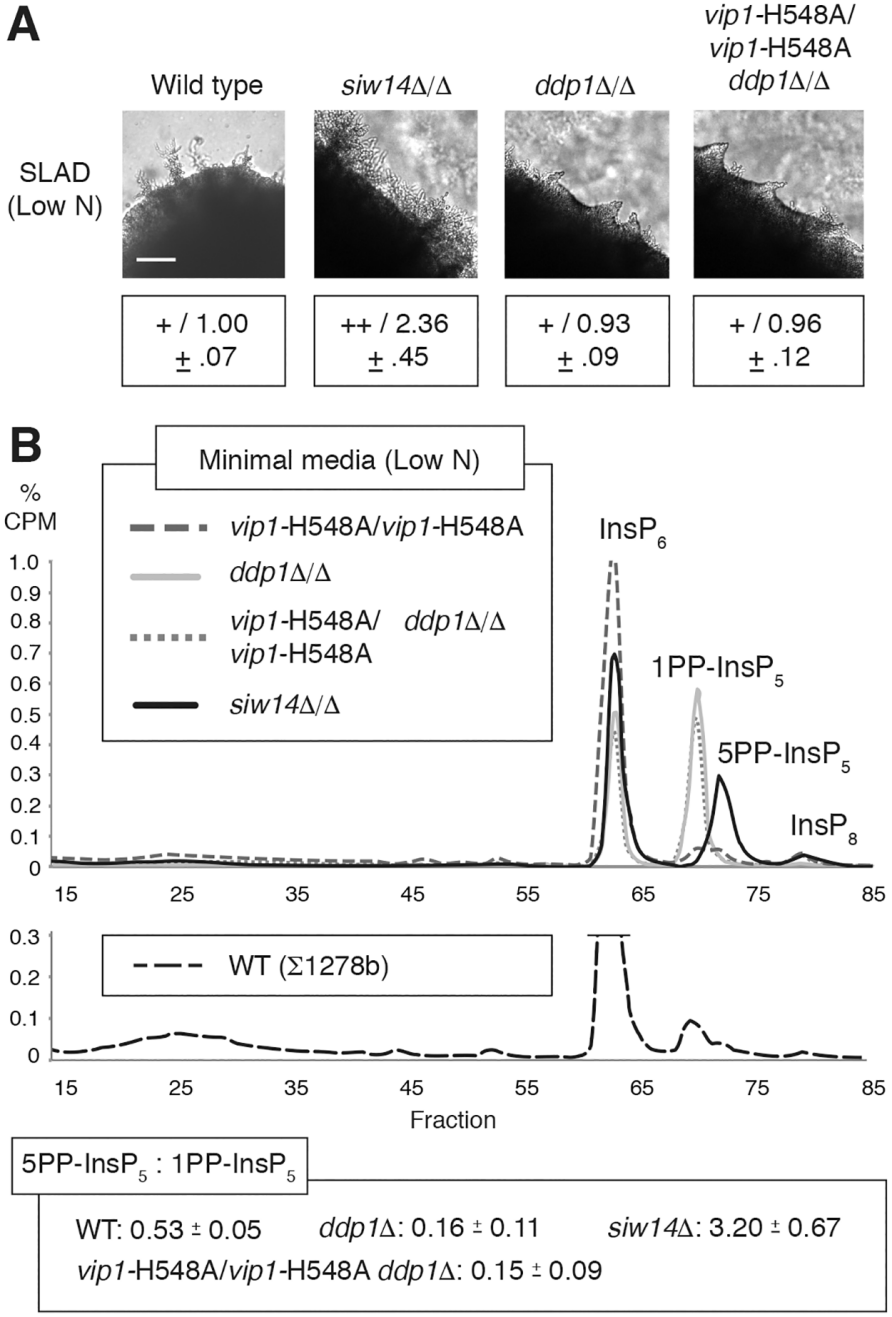
Deletion of the *SIW14* phosphatase gene results in exaggerated pseudohyphal growth. **(A)** Surface pseudohyphal filamentation phenotypes of homozygous diploid strains with the indicated InsP phosphatase mutations. Strains were grown on low-nitrogen SLAD medium. Exaggerated pseudohyphal growth in the *siw14*Δ/Δ mutant is indicated with a “++”; wild-type pseudohypha growth levels are indicated with a “+”. Pseudohyphal growth assays were performed and quantified as described; mean values with standard deviation for three replicates are indicated. Scale bar, 500 μm. **(B)** Representative InsP profiles of mutated InsP phosphatase strains grown in low-nitrogen minimal media for eight hours. The proportional CPM counts are shown to 1% in order to indicate the changes in IP_6_ levels. The InsP trace for a wild-type control strain is shown below the plot. Ratios of 5PP-InsP_5_:1PP-InsP_5_ are indicated as mean with standard deviation. Ratios of InsP_7_ in individual replicates are listed in S7 Table.

Phenotypic analysis of the homozygous diploid *siw14*Δ/Δ deletion mutant indicated exaggerated surface-spread filamentation relative to wild type. The *ddp1*Δ/Δ mutant was wild-type with respect to pseudohyphal growth (Fig 4A). *FLO11* mRNA levels were elevated in the hyperfilamentous *siw14*Δ/Δ mutant relative to wild type under conditions of low nitrogen (S2 Table). InsP profiling of both the *siw14*Δ/Δ and *ddp1*Δ/Δ strains under conditions of nitrogen limitation identified elevated levels of 5PP-InsP_5_ relative to 1PP-InsP_5_ in *siw14*Δ/Δ and increased levels of 1PP-InsP_5_:5PP-InsP_5_ in *ddp1*Δ/Δ (Fig 4B). In both mutants, the reservoir of InsP_6_ was depressed, accounting partially for increases in the respective InsP_7_ isoforms. The scale in Fig 4B has been adjusted to indicate the change in InsP_6_ levels. These results highlight the strong correlation between elevated 5PP-InsP_5_:1PP-InsP_5_ ratios and hyperactive pseudohyphal growth.

To further consider the effect of perturbing levels of the 1PP-InsP_5_ isoform of InsP_7_, we generated a homozygous diploid strain of yeast containing a phosphatase-defective allele of *VIP1* and a deletion of *DDP1*. As Ddp1p and Vip1p are both capable of removing the β-phosphate group from position one of PP-InsPs, we expected this mutant to produce highly elevated levels of 1PP-InsP_5_. InsP profiling of the *vip1*-H548A/H548A *ddp1*Δ/Δ double mutant under conditions of nitrogen limitation identified a trace very similar to that observed in *ddp1*Δ/Δ under identical conditions. The double mutant exhibited wild-type levels of pseudohyphal growth, also similar to the phenotype of *ddp1*Δ/Δ (Fig 4A and S1 Fig).

### Inositol pyrophosphate kinase overexpression driving elevated 5PP-InsP_5_ levels results in elevated pseudohyphal growth

Our results thus far indicate that InsP signaling is required for pseudohyphal growth and that InsP profiles, particularly with respect to the relative levels of 5PP-InsP_5_ and other inositol pyrophosphates, change in correlation with the degree of pseudohyphal growth. To establish these points more strongly, we sought to further perturb InsP signaling towards the production of specific InsP species for analysis of pseudohyphal growth. Because of the charged phosphate groups decorating the *myo*-inositol backbone, InsPs cannot be exogenously added for efficient uptake in yeast. Consequently, we utilized gene overexpression and deletion to impact InsP signaling.

**Fig 5:**
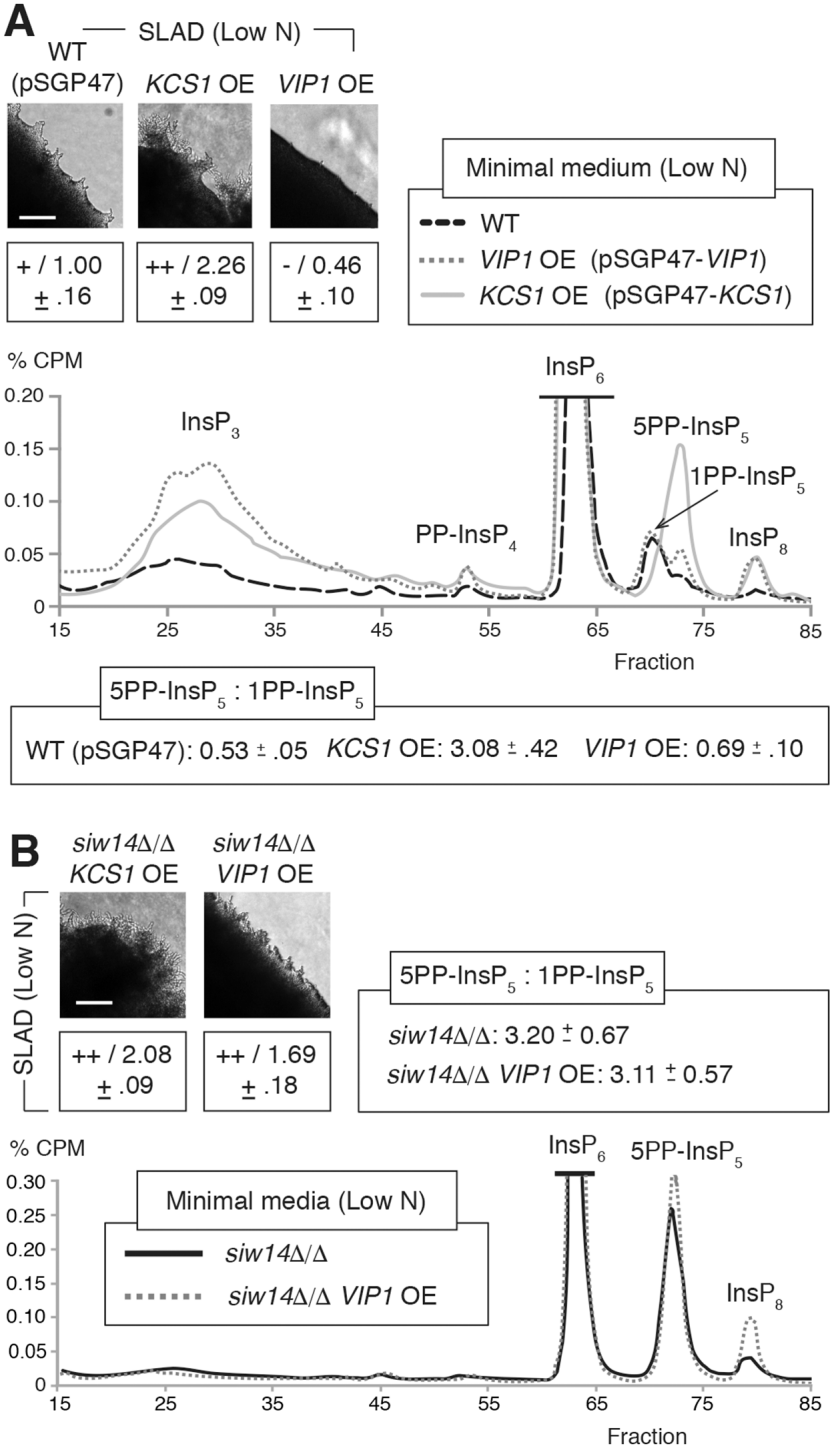
Overexpression mutants with elevated levels of 5PP-InsP_5_ relative to other inositol pyrophosphates exhibit exaggerated pseudohyphal growth. **(A)** Representative InsP profiles of strains overexpressing *VIP1* and *KCS1* grown for eight hours in low-nitrogen minimal media. The ratio of InsP_7_ isoforms is indicate as mean values with standard deviations shown. InsP_7_ ratios in independent replicates are shown in S7 Table. **(B)** Representative InsP profile of *VIP1* overexpression in a *siw14*Δ/Δ background strain grown in low nitrogen media; a representative InsP profile for *siw14*Δ/Δ is included also. The InsP trace of the wild-type strain for this analysis is shown in panel A; all strains were grown under identical conditions. Pseudohyphal growth phenotypes are provided for the indicated mutants. Filamentation was quantified as described in Materials and Methods; mean values with standard deviation are indicated. Scale bars, 500 μm.

To overexpress *KCS1* and *VIP1*, we cloned each gene into a high copy vector such that the coding sequence was expressed under transcriptional control of the *ADH2* promoter. Relative to wild type, *KCS1* overexpression under conditions of nitrogen limitation resulted in elevated levels of InsP_3_, 5PP-InsP_5_, and InsP_8_, with decreased levels of 1PP-InsP_5_ (Fig 5A). *KCS1* overexpression mutants exhibited a ratio of 5PP-InsP_5_ to 1PP-InsP_5_ of over 2, while *VIP1* overexpression resulted in a ratio of the 5PP-InsP_5_:1PP-InsP_5_ isoforms of roughly 0.5. Analysis of pseudohyphal growth phenotypes in the mutants indicated elevated surface-spread filamentation in the *KCS1* overexpression mutant under conditions of nitrogen limitation, while the *VIP1* overexpression strain exhibited decreased filamentation relative to wild type (Fig 5A and S2 Fig). Deletion of *SIW14*, *IPK1*, and *VIP1* in a strain carrying the pSGP47-*KCS1* plasmid overexpressing *KCS1* resulted in hyperfilamentous growth, resembling the background *KCS1* overexpression mutant (Fig 5B and S2 Fig). Deletion of *KCS1* in a strain overexpressing *VIP1* did not change the hypofilamentous phenotype of the parent *VIP1* overexpression mutant (S2 Fig). Deletion of *SIW14* and *IPK1*, however, did alter that phenotype, resulting in exaggerated pseudohyphal filamentation relative to wild type (Fig 5B and S2 Fig). In contrast to the background *VIP1* overexpression strain carrying the pSGP47-*VIP1* construct, InsP profiling of *siw14*Δ/Δ with pSGP47-*VIP1* grown in low-nitrogen media indicated strongly elevated levels of 5PP-InsP_5_ relative to 1PP-InsP_5_ (Fig 5B). An elevated ratio of 5PP-InsP_5_:1PP-InsP_5_ was also evident in the *siw14*Δ/Δ homozygous diploid strain, consistent with the hyper-filamentous phenotype of both mutants. *FLO11* mRNA levels were elevated in the hyperfilamentous *siw14*Δ/Δ strains, with particularly high levels evident in *siw14*Δ/Δ backgrounds carrying either pSGP47-*KCS1* or pSGP47-*VIP1* (S2 Table).

### Perturbation of InsP signaling bypasses nitrogen limitation to allow pseudohyphal growth

**Fig 6:**
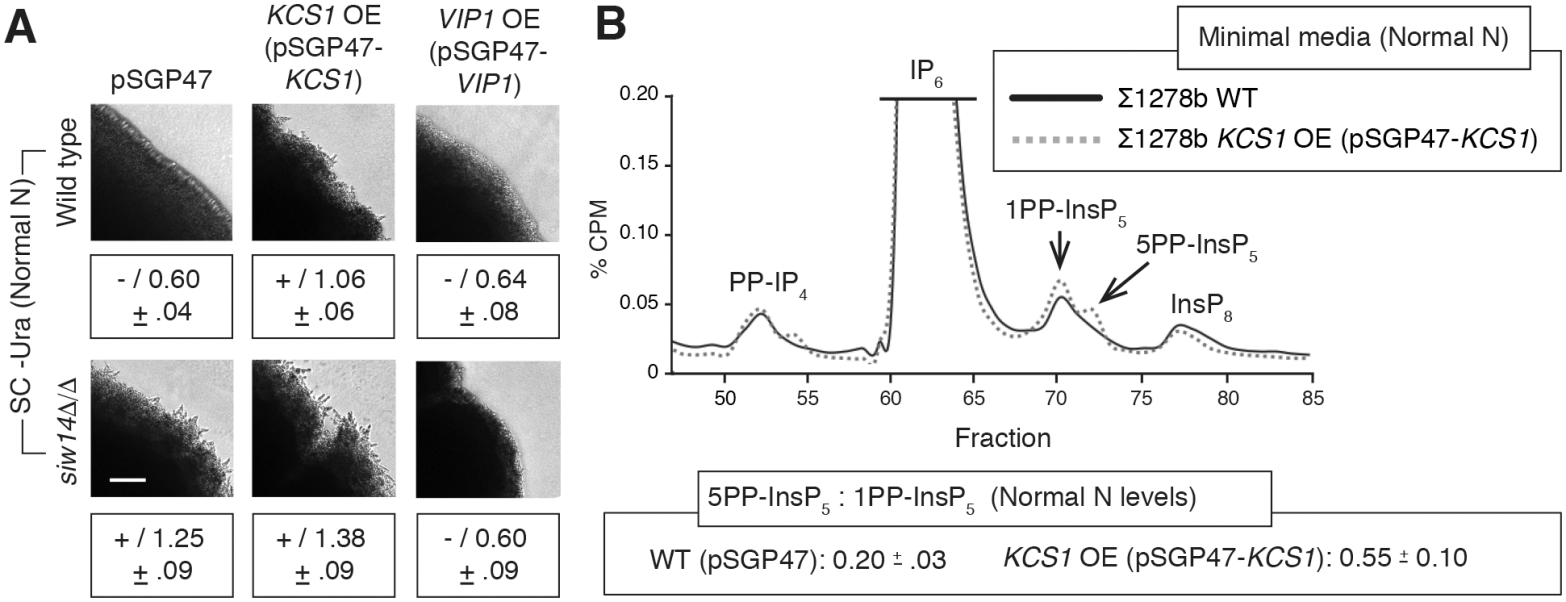
InsP signaling mutants can uncouple the pseudohyphal response from growth in nitrogen-limiting conditions. **(A)** Pseudohyphal growth phenotypes for InsP signaling mutants grown on standard media with normal levels of ammonium sulfate. The wild-type strain carries the empty pSGP47 vector so that all strains can be cultured uniformly. The wild-type filamentous strain exhibits no pseudohyphal growth under these conditions (“-”). To maintain consistency with other figures, pseudohyphal growth was measured here relative to the corresponding wild-type strain grown under low-nitrogen conditions that induce filamentation. By this comparison, as expected, the wild-type strain under normal nitrogen conditions does not exhibit pseudohyphal filamentation with a colony circumference ratio of roughly 0.6 compared against levels observed in low-nitrogen media. Scale bar, 500 μm. **(B)** Representative InsP profiles of the indicated strains grown in media with normal levels of ammonium sulfate. Fractions are indicated on the X-axis, with percent CPM shown on the Y-axis. Mean ratios of 5PP-InsP_5_:1PP-InsP_5_ with standard deviations are shown for the analyzed strains.

To assess the degree to which InsP signaling can impact pseudohyphal growth independently of other stimuli, we analyzed a set of strains with mutations affecting InsP kinases and/or phosphatases on media with normal levels of ammonium sulfate. In the presence of media with nitrogen levels that are repressive for filamentation, overexpression of *KCS1* and deletion of *SIW14*, individually and in combination, resulted in surface-spread pseudohyphal growth (Fig 6A). InsP profiling of a *KCS1* overexpression strain grown in media with normal nitrogen levels indicates two peaks corresponding to the 5PP-InsP_5_ and 1PP-InsP_5_ isoforms of InsP_7_ (Fig 6B). It is notable that the two InsP_7_ isoforms are present in this *KCS1* overexpression mutant even under conditions with normal nitrogen levels, in contrast to the corresponding wild-type strain under identical conditions.

### Pseudohyphal growth kinases are required for wild-type InsP levels

Since kinases and phosphatases in the InsP biosynthesis pathway are essential for wild-type pseudohyphal growth, we investigated the possibility that signaling pathways required for pseudohyphal growth may regulate levels of InsP species. For this study, we focused on the Snf1p kinase pathway and Kss1p and Fus3p MAPKs, as we previously identified InsP kinases as being differentially phosphorylated in strains with kinase-defective alleles of *SNF1* and MAPKs [43]. Deletion of *TPK2*, encoding a catalytic subunit of protein kinase A required for pseudohyphal growth, did not substantially affect InsP levels under conditions of nitrogen limitation.

**Fig 7:**
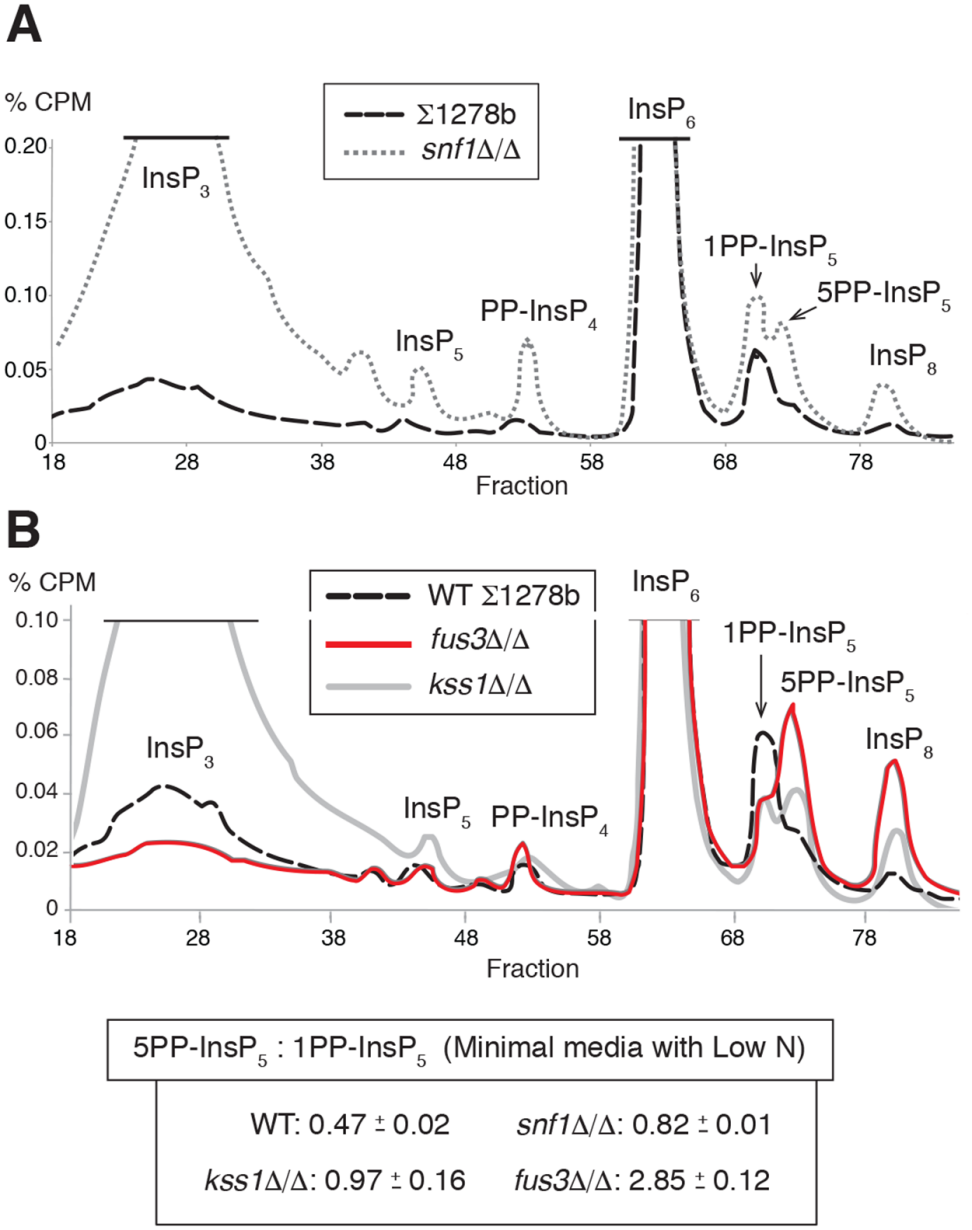
The pseudohyphal growth regulatory kinases Snf1p, Kss1p, and Fus3p are required for wild-type InsP signaling. **(A)** Representative InsP profiles of the wild-type filamentous Σ1278b strain and an otherwise isogenic *snf1*Δ/Δ mutant grown in low-nitrogen media. **(B)** Representative InsP profiles are indicated for *kss1*Δ/Δ and *fus3*Δ/Δ mutants grown in low-nitrogen media; an InsP profile of the wild-type Σ1278b strain under identical growth conditions is provided for comparison. Mean ratios of the InsP_7_ isoforms are shown with standard deviation. InsP_7_ ratios of independent replicates are provided in S7 Table.

The homozygous diploid *snf1*Δ/Δ strain is defective in pseudohyphal growth with no filamentation evident in low-nitrogen media [11, 18, 46]. InsP profiling of *snf1*Δ/Δ indicated elevated levels of InsP_3_, InsP_5_, PP-InsP_4_, the InsP_7_ isoforms, and InsP_8_ (Fig 7A). The highly elevated levels of InsP_3_ and the absence of a predominance of the 5PP-InsP_5_ isoform relative to 1PP-InsP_5_ and InsP_8_ are particularly notable. As evidenced in the *snf1*Δ/Δ mutant, a homozygous *kss1*Δ/Δ strain displayed elevated levels of InsP_3_. The *kss1*Δ/Δ mutant is hypofilamentous and showed roughly comparable levels of the 1PP-InsP_5_ and 5PP-InsP_5_ isoforms (Fig 7B), with elevated levels of InsP_8_. The mating pathway MAPK Fus3 negatively regulates pseudohyphal growth by phosphorylating and targeting the transcription factor Tec1p for destruction; its deletion results in exaggerated invasive and surface-spread filamentation [18, 47]. Consistent with the pattern observed in other mutants with exaggerated pseudohyphal growth, the hyperfilamentous *fus3*Δ/Δ mutant exhibited an elevated ratio of 5PP-InsP_5_ to 1PP-InsP_5_ (Fig 7B). InsP_8_ levels were also increased in *fus3*Δ/Δ.

To determine if InsP signaling in turn impacts MAPK pathway activity, we assessed the phosphorylation state of Kss1p in strains singly deleted for the InsP pathway genes *PLC1*, *IPK1*, *VIP1*, and *KCS1* (S3 Fig). Kss1p phosphorylation was not strikingly affected in any of these mutants, suggesting that the MAPK pathway may not be acted upon by InsP signaling; the possibility still exists, however, that MAPK signaling may regulate InsP-based signaling.

## Discussion

InsP signaling has been studied for over two decades, but its functional significance has not been fully elucidated. Owing partially to the labor involved, InsPs have not been profiled over a broadly representative spectrum of growth conditions. Here, we used the genetic workhorse *Saccharomyces cerevisiae* to profile InsP levels under conditions of nitrogen limitation in a filamentous background, and our results indicate a new role for InsP signaling in the pseudohyphal growth response. Perturbation of the InsP biosynthesis pathway altered pseudohyphal growth, and *in vivo* measurement of InsP levels in these mutants identified characteristic profiles, particularly with respect to the inositol pyrophosphates, that were predictive of pseudohyphal growth states. Mutation of genes encoding InsP kinases and phosphatases enabled pseudohyphal growth in the absence of nitrogen limitation. InsP profiles were substantially altered in mutants deleted of *SNF1*, *KSS1*, and *FUS3*, suggesting that these well studied kinase regulators of pseudohyphal growth contribute to the establishment and/or maintenance of wild-type InsP levels under conditions of nitrogen limitation.

Considered collectively, the data suggest the ratio of the InsP_7_ isoforms is indicative of the pseudohyphal growth state. Elevated levels of 5PP-InsP_5_ relative to the levels of 1PP-InsP_5_ were evident in strains that showed exaggerated pseudohyphal growth. This pattern held consistent under conditions of nitrogen limitation in homozygous diploid *vip1*Δ/Δ, *vip1*-D487A/D487A, *siw14*Δ/Δ, *KCS1* overexpression, and *fus3*Δ/Δ strains. Further, in low-nitrogen media, overexpression of *VIP1* in a strain deleted of *SIW14* resulted in elevated levels of 5PP-InsP_5_ and hyperfilamentation. Collectively, the ratios of InsP_7_ isoforms in these hyperfilamentous mutants is distinct from corresponding ratios in wild-type strains by an independent samples T-test (*p*<0.001) (S4A Fig). This set of hyperfilamentous mutants exhibit 5PP-InsP_5_ to 1PP-InsP_5_ ratios that are also distinct from those observed in hypofilamentous mutants (*kcs1*Δ/Δ, *VIP1* overexpression, *snf1*Δ/Δ, and *kss1*Δ/Δ mutants) (*p*<0.001) (S4B Fig). It is interesting that wild-type pseudohyphal growth did not indicate highly elevated levels of 5PP-InsP_5_ relative to 1PP-InsP_5_ and InsP_8_, but instead was characterized by decreased InsP_5_ and elevated InsP_7_ isoforms, with 1PP-InsP_5_ showing the greater increase. It should also be noted that exaggerated levels of PP-InsP_4_ and 1,5(PP)_2_-InsP_3_ in *ipk1*Δ/Δ promote pseudohyphal growth, although the effects may be observed only upon removal of the higher energy IP_7_ and IP_8_ pyrophosphates, as we did not observe consistent changes in PP-InsP_4_ and 1,5(PP)_2_-InsP_3_ levels over the mutants we analyzed. Additional InsP profiling in a larger mutant set will likely be necessary to refine the characteristic InsP signatures, but the data are consistent with the notion that InsP profiles are a predictive indicator of mutant pseudohyphal growth states.

These results highlight the importance of inositol pyrophosphates, and particularly InsP_7_ isoforms, in the pseudohyphal growth response. It is notable that two peaks corresponding to the InsP_7_ isoforms 5PP-InsP_5_ and 1PP-InsP_5_ are present in profiles of strains grown in low-nitrogen media. Previous analyses of InsP profiles in yeast by HPLC-based fractionation have presented a single peak corresponding to InsP_7_: however, these studies were not undertaken using nitrogen-limiting growth media, nor using the 15-minute extended separation gradient we employed here. In fact, our InsP profiles of the wild-type Σ1278b strain under normal growth conditions do indicate a single InsP_7_ peak. This single peak had previously been identified from analyses of non-filamentous yeast as 5PP-InsP_5_ [26, 33, 45]; however, our fractionation profiles indicate that this peak corresponds to 1PP-InsP_5_. Several lines of evidence support this conclusion. First, InsP profiles of *kcs1*Δ/Δ grown on low-nitrogen media identify a small peak aligning with the one observed in profiles of a wild-type strain under normal growth conditions. This peak most likely corresponds to Vip1p-produced 1PP-InsP_5_, as Vip1p is capable of pyrophosphorylating InsP_6_ in a strain deleted of *KCS1*. Second, profiles of *vip1*Δ/Δ indicate a very large peak aligning with the later InsP_7_ isoform fraction, which, by similar logic, likely represents Kcs1p-produced 5PP-InsP_5_. Further, an identical peak is observed in profiles of *siw14*Δ/Δ in which 5PP-InsP_5_ accumulates, as this isomer cannot be dephosphorylated in the absence of *SIW14* [32]. Thus, the data indicate that we can efficiently separate InsP_7_ isomers in yeast under low nitrogen conditions through an extended gradient, with 1PP-InsP_5_ fractionating just ahead of 5PP-InsP_5_.

Beyond its relevance to yeast pseudohyphal growth, this study is also informative in considering the role of InsPs in the cellular response to nitrogen limitation. Filamentous and non-filamentous strains grown in media with reduced ammonium sulfate exhibit many similar changes in InsP levels, including elevated levels of pyrophosphorylated InsP_7_ isoforms as discussed above. These data suggest that the transition to conditions of decreased nitrogen availability necessitates the increased accumulation of both InsP_7_ isoforms, making them detectable by labeling and fractionation protocols. It is additionally interesting that overexpression of *KCS1* in normal media results in InsP fractionation peaks corresponding to 5PP-InsP_5_ and 1PP-InsP_5_, mimicking the profile observed in strains grown under conditions of low nitrogen with associated filamentation. Thus, InsP levels can be manipulated to uncouple pseudohyphal growth from nitrogen limitation, and, further, inositol pyrophosphate abundance may be an intracellular signal of nitrogen conditions, functioning as part of the cellular response in yeast to manage nitrogen limitation.

InsP profiles in this study as well as previous phosphoproteomic data are consistent with a role for AMPK and MAPK signaling in regulating InsP levels under conditions of nitrogen limitation, and the results here provide the first evidence that AMPK and MAPK signaling is required for wild-type levels of InsPs. Interestingly, inositol polyphosphate multikinase has previously been demonstrated to be capable of binding AMPK [48]. These signaling modules are established regulators of cellular responses to nutrient levels, and functions for Snf1p, Kss1p, and Fus3p in controlling InsP levels would provide an important point of crosstalk between classic cell signaling pathways and metabolic second messengers. In sum, this work further establishes the foundation for additional studies of the regulation and downstream effectors of InsP signaling in cellular stress responses and pseudohyphal growth.

## Materials and methods

### Strains, plasmids, and media

Strains used in this study are listed in S3 Table. Filamentous yeast strains were derived from the genetic background Σ1278b [5, 49]. Haploid strains were derived from HLY337 and Y825 [50, 51]. Non-filamentous strains were derived from BY4743 [52]. Standard protocols for proper growth and maintenance of yeast have been described previously [53, 54]. DNA was introduced into yeast by standard protocols involving lithium acetate treatment and heat shock [55].

Plasmids for *ADH2*-based overexpression [56] were derived from pSGP47. The pSGP47 vector contains the *S*. *cerevisiae* 2μ origin of replication, resulting in roughly 50 copies per cell. Transcript levels of *KCS1* and *VIP1* from these overexpression vectors were assessed under normal and low-nitrogen conditions, and the results are presented in S4 Table. The plasmids enable strong overexpression of the *KCS1* and *VIP1* genes, particularly under conditions of low nitrogen.

*S*. *cerevisiae* strains were cultured on YPD (1% yeast extract, 2% peptone, 2% glucose) or Synthetic Complete (SC) media (0.67% Yeast Nitrogen Base without amino acids, 2% glucose, and 0.2% of the appropriate amino acid drop-out mix). Nitrogen limitation conditions for pseudohyphal growth phenotypic assays were achieved using Synthetic Low Ammonium Dextrose (SLAD) media (0.17% Yeast Nitrogen Base without amino acids and without ammonium sulfate, 2% glucose, 50 μM ammonium sulfate, and appropriate amino acids as necessary). For inositol polyphosphate profiles, yeast cultures were grown in minimal media lacking inositol (0.17% Yeast Nitrogen Base without ammonium sulfate, amino acids, and inositol, 2% glucose, 0.5% ammonium sulfate, and appropriate amino acids) and as needed were subsequently grown in SLAD-inositol media (0.17% Yeast Nitrogen Base without amino acids, ammonium sulfate, and inositol, 2% glucose, 50 μM ammonium sulfate, and any required amino acids).

### Gene deletions and integrated point mutations

Gene deletions were generated by one-step PCR-based replacement using either KanMX6 or HphMX6 drug-resistance cassettes of pFA6a-KanMX6 or pAG32 [57]. Gene replacement was verified by PCR-based methods as described [58]. Diploid mutants were typically constructed by deleting each desired gene in haploid backgrounds of opposite mating type and subsequently mating the strains, selecting for complementary auxotrophies. The *kss1*Δ/Δ and *fus3*Δ/Δ mutants were generated in a diploid cell where each gene copy was deleted by allelic replacement with the indicated drug resistance markers [57]. Overexpression plasmids were constructed by ligation-independent cloning using the parent pSGP47 vector [59, 60]. Integrated point mutations were generated using the *URA3*-flip-out method as previously described [61, 62].

### Filamentous growth assays

For invasive growth assays, cultures of haploid strains were grown overnight with shaking in YPD media at 30°C, harvested, and adjusted to an optical density of 1 at 600 nm in sterile water. A 5 μl aliquot of the culture was spotted onto YPD plates and allowed to invade the agar for two days. Plates were photographed for surface growth and then gently washed with a light stream of water and photographed again to visualize invasive growth [49, 58, 63]. The degree of invasive growth was quantified as the mean pixel intensity of the washed spot relative to its pre-washed image using the program ImageJ, as described previously [20]. Triplicate replicates were assayed for each haploid strain tested, and results for each independent replicate are provided in S5 Table. Results were binned as follows: hypofilamentous, less than 0.5; wild-type, between 0.5 and 0.7; hyperfilamentous, greater than 0.7.

For surface spread assays, diploid strains were grown overnight in appropriate SC-dropout media. Saturated cultures were spread onto SLAD medium and incubated at 30°C for four days [5, 50]. Colonies were imaged using an upright Nikon Eclipse 80i microscope with CoolSnap ES2 CCD (Photometrics). Images were acquired using MetaMorph software (Molecular Devices). The extent of surface-spread pseudohyphal growth was quantified using the approach of Ryan *et al*. [19] with minor modifications. In brief, the circumference of a defined area of a colony is measured using the NeuronJ program, and the value is compared against the corresponding circumference of the same defined area of a wild-type strain colony under conditions of low nitrogen. The ratio is presented for three replicates (S6 Table), and the average and standard deviation is indicated. Mean ratios of greater than 1.5 are indicated as hyperfilamentous (++), and mean ratios of less than 0.75 are indicated as hypofilamentous (-).

### Expression analysis of *FLO11*, *KCS1*, and *VIP1*

Single colonies were inoculated in 4 ml appropriate medium (YPD or SC-Ura). Overnight cultures were diluted 1:1000 in 5 ml YPD or SC-URA and let grow for 16–20 hours. The cells were harvested at 3000*g* for 5 minutes and washed twice with water. If no nitrogen stress was utilized, the cells were either stored at -80°C or subjected to RNA extraction right away. If nitrogen stress was utilized, the cells were resuspended in 5 ml SLAD medium and incubated at 30°C for 8 hours. After incubation, the cells were harvested and washed twice with water. The cells were either stored at -80°C or subjected to RNA extraction right away. RNA extraction was done using the RiboPure-Yeast kit (Invitrogen) according to manufacturer’s directions. The amount of RNA isolated was determined using a NanoDrop spectrophotometer/fluorometer. For each sample, 1 μg of RNA was converted to cDNA using the Radiant cDNA Synthesis Kit, 1-Step. The resulting cDNA was diluted 1:100 and 2 μl of the diluted cDNA was used as a template for qPCR. qPCR mixes were prepared using the Radiant Green Hi-ROX qPCR kit and run with an Applied Biosystems StepOne Plus qPCR machine. The relative amounts of *FLO11*, *KCS1*, and *VIP1* expression were calculated using the double delta Ct method with *ACT1* as the reference gene.

Primers used for qRT-PCR are as follows: *ACT1*-qPCR-F: CTGCCGGTATTGACCAAACT; *ACT1*-qPCR-R: CGGTGATTTCCTTTTGCATT; *FLO11*-qPCR-FWD3: GTTGTTTCGCCAGCGGAGTT; *FLO11*-qPCR-RVS3: CTACCACCCCTGTCCGACG; *KCS1*-qPCR-FWD3: GCAATAATGGCGGGTCCGTG; *KCS1*-qPCR-RVS3: TGCGCCACGTGTTTATTGGG; *VIP1*-qPCR-FWD1: AGAGCTCTTTTTGGGGCCGA; *VIP1*-qPCR-RVS1: GTGGGGGAGCTTCCTTACCC.

### Inositol polyphosphate profiling

HPLC analysis of InsP levels was conducted as previously described [45] with the following modifications. In brief, yeast cultures were grown overnight at 30°C with shaking until fully saturated in rich media. Subsequently, 5 μl of the saturated culture was added to 5 ml of minimal media lacking inositol supplemented with 25 μl of *myo*[1,2-^3^H]inositol 1 mCi/ml 30 Ci/mmol (American Radiolabeled Chemicals cat. no. ART02611MC) and allowed to grow overnight at 30°C with shaking at 250 rpm until reaching an OD_600_ of 0.9. Cells were then either harvested and frozen at -80°C until further use or harvested and washed with water prior to being suspended in 5 ml of SLAD-inositol media; this culture was grown for 8 hours at 30°C at 250 rpm. These cultures were harvested and frozen at -80°C until further use. Inositol polyphosphates were extracted by suspending the thawed pellet in 300 μl of 1M perchloric acid with 3mM EDTA and bead beating at 4°C for five minutes. Samples were spun down, and the resulting supernatant was saved and neutralized with the addition of 1 M potassium carbonate and 3 mM EDTA until a pH of 6.0–8.0 was reached. The mixture was allowed to sit on ice for two hours. The sample was again centrifuged, and the clear supernatant was analyzed by HPLC (Hewlett Packard Series 1100) connected to a Partisphere 5 μm SAX cartridge column 125 x 4.6 mm (HiCHROM cat. no. 4621–0505). Inositol polyphosphates were eluted from the column with a gradient from mixing buffer A (1 mM EDTA) and buffer B (1.3 M (NH_4_)_2_HPO_4_, 1 mM EDTA, pH to 3.8 with H_3_PO_4_). The gradient was as follows: 0–5 min, 0% buffer B; 5–10 min, 0–10% buffer B; 10–90 min, 10–100% Buffer B; 90–100 min, 100% Buffer B; 100–101 min, 0% buffer B; 101–110 min 0% buffer B. The gradient was run at a flow rate of 1 ml/min and one-1ml fractions were collected every minute for the first 90 minutes. Fractions were mixed with 4 ml of Ultima-Flo AP liquid scintillation cocktail (Perkin-Elmer cat. no. 6013599) and counted using a scintillation counter.

## Supporting information

S1 Fig Cell morphology of IP kinase and phosphatase mutants under conditions that induce pseudohyphal growth. **(A)** The indicated strains deleted for genes encoding IP kinases were grown in low-nitrogen media along with the wild-type Σ1278b parent strain. Cells were scraped from a colony after growth in low-nitrogen SLAD medium, and the cells were suspended in solution prior to DIC imaging. Cells with length-to-width ratios greater than 2.0 are highlighted with asterisks. Scale bar, 5 μm. **(B)** Cell morphology of indicated strains mutated for genes encoding IP phosphatases. Cells were grown and imaged as above. Scale bar, 5 μm.
(TIF)

S2 Fig Filamentous growth phenotypes of overexpression mutants perturbing IP biosynthesis. **(A)** Cells of the indicated strains were scraped from colonies grown on low-nitrogen SLAD medium and imaged by DIC. Cells with length-to-width ratios greater than 2.0 are highlighted with asterisks. Scale bar, 5 μm. **(B)** Surface-spread filamentation phenotypes of indicated mutant strains. “++” represents hyperfilamentous growth, and “-” indicates an absence of pseudohyphal filamentation. Scale bar, 500 μm.
(TIF)

S3 Fig Kss1p phosphorylation in InsP pathway mutants. Cells were grown overnight in YPD and 30°C. Cells were sub-cultured into either YPD or YP-Gal medium and grown for 6 hours. Protein extracts were made by TCA precipitation and separated by 10% SDS-PAGE. Proteins were transferred to nitrocellulose membrane and blotted with either p44/42 antibody (Cell Signaling Technology, Danvers, MA; #4370) or Kss1 antibody (Santa Cruz Biotechnology, Santa Cruz, CA; #6775) or Pgk1 antibody (Life Technologies; Camarillo, CA; #459250) as indicated. For secondary antibodies (goat anti-mouse IgG–HRP, Bio-Rad Laboratories, Hercules, CA; #170–6516; goat anti-rabbit IgG-HRP, Jackson ImmunoResearch Laboratories, Inc., West Grove, PA; #111-035-144) were used. Membranes were developed using Western Bright kit from Advansta Inc. (Menlo Park, CA; LPS #K-12045-D20) and imaged using Imagelab software (Biorad Inc.). The *ste11*Δ mutant is a control for diminished Kss1p phosphorylation. Levels of phosphorylated Kss1p relative to wild-type were estimated by densitometry with normalization to the Pgk1p loading control.
(TIF)

S4 Fig Hyperfilamentous mutants exhibit 5PP-InsP_5_:1PP-InsP_5_ ratios distinct from those observed in wild-type and hypofilamentous strains. **(A)** By independent samples T-test, the difference in ratios of the InsP_7_ isoforms between hyperfilamentous mutants analyzed in this study (*vip1*Δ/Δ, *vip1*-D487A/D487A, *siw14*Δ/Δ, *KCS1* overexpression, *siw14*Δ/Δ *VIP1* overexpression double mutants, and *fus3*Δ/Δ strains) and wild-type control strains is statistically significant (*p*<0.001). **(B)** Hyperfilamentous mutants indicated above also exhibit a statistically significant difference (*p*<0.001) in 5PP-InsP_5_:1PP-InsP_5_ ratios from hypofilamentous mutants (*kcs1*Δ/Δ, *VIP1* overexpression, *snf1*Δ/Δ, and *kss1*Δ/Δ mutants) analyzed in this study.
(TIF)

S1 Table The pseudohyphal growth kinases Snf1p and Kss1p are required for wild-type phosphorylation of IP kinases. (RTF)

S2 Table *FLO11* mRNA levels in InsP phosphatase mutants. (RTF)

S3 Table List of strains used in this study. (RTF)

S4 Table mRNA levels of over-expressed *KCS1* and *VIP1* in high-copy vectors with the *ADH2* promoter. (RTF)

S5 Table Invasive growth assay datasets. (RTF)

S6 Table Pseudohyphal filament circumference of independent replicate colonies. (RTF)

S7 Table Ratio of InsP_7_ isoforms in independent replicates for indicated strains. (RTF)

S1 Dataset Percent CPM values for separated fractions plotted in InsP traces. (XLSX)

## References

[1] Mitchell AP. Dimorphism and virulence in *Candida albicans*. Curr Opin Microbiol. 1998; 1(6):687–92. 10066539 10.1016/s1369-5274(98)80116-1

[2] Madhani HD, Fink GR. The control of filamentous differentiation and virulence in fungi. Trends Cell Biol. 1998; 8:348–53. 9728395 10.1016/s0962-8924(98)01298-7

[3] Berman J, Sudbery PE. *Candida albicans*: a molecular revolution built on lessons from budding yeast. Nat Rev Genet. 2002; 3(12):918–30. doi: 10.1038/nrg948 12459722 10.1038/nrg948

[4] Okagaki LH, Strain AK, Nielsen JN, Charlier C, Baltes NJ, Chretien F, et al Cryptococcal cell morphology affects host cell interactions and pathogenicity. PLoS Pathog. 2010; 6(6):e1000953 doi: 10.1371/journal.ppat.1000953 20585559 10.1371/journal.ppat.1000953 PMC2887476

[5] Gimeno CJ, Ljungdahl PO, Styles CA, Fink GR. Unipolar cell divisions in the yeast *S*. *cerevisiae* lead to filamentous growth: regulation by starvation and RAS. Cell. 1992; 68(6):1077–90. 1547504 10.1016/0092-8674(92)90079-r

[6] Lo WS, Dranginis AM. FLO11, a yeast gene related to the STA genes, encodes a novel cell surface flocculin. J Bacteriol. 1996; 178:7144–51. 8955395 10.1128/jb.178.24.7144-7151.1996 PMC178626

[7] Cullen PJ, Sprague GF. Glucose depletion causes haploid invasive growth in yeast. Proc Natl Acad Sci U S A. 2000; 97:13461–3. 11095711 10.1073/pnas.240345197 PMC17625

[8] Lo HJ, Kohler J, DiDomenico B, Loebenberg D, Cacciapuoti A, Fink GR. Nonfilamentous *C*. *albicans* mutants are avirulent. Cell. 1997; 90:939–49. 9298905 10.1016/s0092-8674(00)80358-x

[9] Saville SP, Lazzell AL, Monteagudo C, Lopez-Ribot JL. Engineered control of cell morphology in vivo reveals distinct roles for yeast and filamentous forms of *Candida albicans* during infection. Eukaryot Cell. 2003; 2(5):1053–60. doi: 10.1128/EC.2.5.1053-1060.2003 14555488 10.1128/EC.2.5.1053-1060.2003 PMC219382

[10] Celenza JL, Carlson M. Cloning and genetic mapping of SNF1, a gene required for expression of glucose-repressible genes in *Saccharomyces cerevisiae*. Mol Cell Biol. 1984; 4(1):49–53. 6366512 10.1128/mcb.4.1.49-53.1984 PMC368656

[11] Vyas VK, Kuchin S, Berkey CD, Carlson M. Snf1 kinases with different beta-subunit isoforms play distinct roles in regulating haploid invasive growth. Mol Cell Biol. 2003; 23(4):1341–8. doi: 10.1128/MCB.23.4.1341-1348.2003 12556493 10.1128/MCB.23.4.1341-1348.2003 PMC141157

[12] Liu H, Styles CA, Fink GR. Elements of the yeast pheromone response pathway required for filamentous growth of diploids. Science. 1993; 262:1741–4. 8259520 10.1126/science.8259520

[13] Cook JG, Bardwell L, Kron SJ, Thorner J. Two novel targets of the MAP kinase Kss1 are negative regulators of invasive growth in the yeast *Saccharomyces cerevisiae*. Genes Dev. 1996; 10(22):2831–48. 8918885 10.1101/gad.10.22.2831

[14] Robertson LS, Fink GR. The three yeast A kinases have specific signaling functions in pseudohyphal growth. Proc Natl Acad Sci U S A. 1998; 95(23):13783–7. 9811878 10.1073/pnas.95.23.13783 PMC24897

[15] Pan X, Heitman J. Cyclic AMP-dependent protein kinase regulates pseudohyphal differentiation in *Saccharomyces cerevisiae*. Mol Cell Biol. 1999; 19(7):4874–87. 10373537 10.1128/mcb.19.7.4874 PMC84286

[16] Erdman S, Snyder M. A filamentous growth response mediated by the yeast mating pathway. Genetics. 2001; 159(3):919–28. 11729141 10.1093/genetics/159.3.919 PMC1461863

[17] Cullen PJ, Sprague GF Jr., The regulation of filamentous growth in yeast. Genetics. 2012; 190(1):23–49. doi: 10.1534/genetics.111.127456 22219507 10.1534/genetics.111.127456 PMC3249369

[18] Jin R, Dobry CJ, McCown PJ, Kumar A. Large-scale analysis of yeast filamentous growth by systematic gene disruption and overexpression. Mol Biol Cell. 2008; 19(1):284–96. doi: 10.1091/mbc.E07-05-0519 17989363 10.1091/mbc.E07-05-0519 PMC2174193

[19] Ryan O, Shapiro RS, Kurat CF, Mayhew D, Baryshnikova A, Chin B, et al Global gene deletion analysis exploring yeast filamentous growth. Science. 2012; 337(6100):1353–6. doi: 10.1126/science.1224339 22984072 10.1126/science.1224339

[20] Shively CA, Eckwahl MJ, Dobry CJ, Mellacheruvu D, Nesvizhskii A, Kumar A. Genetic networks inducing invasive growth in *Saccharomyces cerevisiae* identified through systematic genome-wide overexpression. Genetics. 2013; 193(4):1297–310. doi: 10.1534/genetics.112.147876 23410832 10.1534/genetics.112.147876 PMC3606104

[21] Irvine RF, Schell MJ. Back in the water: the return of the inositol phosphates. Nat Rev Mol Cell Biol. 2001; 2(5):327–38. doi: 10.1038/35073015 11331907 10.1038/35073015

[22] Flick JS, Thorner J. Genetic and biochemical characterization of a phosphatidylinositol-specific phospholipase C in Saccharomyces cerevisiae. Mol Cell Biol. 1993; 13(9):5861–76. 8395015 10.1128/mcb.13.9.5861-5876.1993 PMC360334

[23] Streb H, Irvine RF, Berridge MJ, Schulz I. Release of Ca2+ from a nonmitochondrial intracellular store in pancreatic acinar cells by inositol-1,4,5-trisphosphate. Nature. 1983; 306(5938):67–9. 6605482 10.1038/306067a0

[24] Saiardi A, Erdjument-Bromage H, Snowman AM, Tempst P, Snyder SH. Synthesis of diphosphoinositol pentakisphosphate by a newly identified family of higher inositol polyphosphate kinases. Curr Biol. 1999; 9(22):1323–6. 10574768 10.1016/s0960-9822(00)80055-x

[25] Shears SB. Assessing the omnipotence of inositol hexakisphosphate. Cell Signal. 2001; 13(3):151–8. 11282453 10.1016/s0898-6568(01)00129-2

[26] Saiardi A, Caffrey JJ, Snyder SH, Shears SB. The inositol hexakisphosphate kinase family. Catalytic flexibility and function in yeast vacuole biogenesis. J Biol Chem. 2000; 275(32):24686–92. doi: 10.1074/jbc.M002750200 10827188 10.1074/jbc.M002750200

[27] Mulugu S, Bai W, Fridy PC, Bastidas RJ, Otto JC, Dollins DE, et al A conserved family of enzymes that phosphorylate inositol hexakisphosphate. Science. 2007; 316(5821):106–9. doi: 10.1126/science.1139099 17412958 10.1126/science.1139099

[28] Glennon MC, Shears SB. Turnover of inositol pentakisphosphates, inositol hexakisphosphate and diphosphoinositol polyphosphates in primary cultured hepatocytes. Biochem J. 1993; 293 (Pt 2):583–90. 8343137 10.1042/bj2930583 PMC1134401

[29] Menniti FS, Miller RN, Putney JW Jr., Shears SB. Turnover of inositol polyphosphate pyrophosphates in pancreatoma cells. J Biol Chem. 1993; 268(6):3850–6. 8382679

[30] Lonetti A, Szijgyarto Z, Bosch D, Loss O, Azevedo C, Saiardi A. Identification of an evolutionarily conserved family of inorganic polyphosphate endopolyphosphatases. J Biol Chem. 2011; 286(37):31966–74. doi: 10.1074/jbc.M111.266320 21775424 10.1074/jbc.M111.266320 PMC3173201

[31] Pohlmann J, Risse C, Seidel C, Pohlmann T, Jakopec V, Walla E, et al The Vip1 inositol polyphosphate kinase family regulates polarized growth and modulates the microtubule cytoskeleton in fungi. PLoS Genet. 2014; 10(9):e1004586 doi: 10.1371/journal.pgen.1004586 25254656 10.1371/journal.pgen.1004586 PMC4177672

[32] Steidle EA, Chong LS, Wu M, Crooke E, Fiedler D, Resnick AC, et al A Novel Inositol Pyrophosphate Phosphatase in *Saccharomyces cerevisiae*: Siw14 protein selectively cleaves the beta-phosphate from 5-diphosphoinositol pentakisphosphate (5PP-IP5). J Biol Chem. 2016; 291(13):6772–83. doi: 10.1074/jbc.M116.714907 26828065 10.1074/jbc.M116.714907 PMC4807264

[33] York JD, Odom AR, Murphy R, Ives EB, Wente SR. A phospholipase C-dependent inositol polyphosphate kinase pathway required for efficient messenger RNA export. Science. 1999; 285(5424):96–100. 10390371 10.1126/science.285.5424.96

[34] Chakraborty A, Koldobskiy MA, Bello NT, Maxwell M, Potter JJ, Juluri KR, et al Inositol pyrophosphates inhibit Akt signaling, thereby regulating insulin sensitivity and weight gain. Cell. 2010; 143(6):897–910. doi: 10.1016/j.cell.2010.11.032 21145457 10.1016/j.cell.2010.11.032 PMC3052691

[35] Monserrate JP, York JD. Inositol phosphate synthesis and the nuclear processes they affect. Curr Opin Cell Biol. 2010;22(3):365–73. doi: 10.1016/j.ceb.2010.03.006 20359876 10.1016/j.ceb.2010.03.006

[36] Burton A, Azevedo C, Andreassi C, Riccio A, Saiardi A. Inositol pyrophosphates regulate JMJD2C-dependent histone demethylation. Proc Natl Acad Sci U S A. 2013; 110(47):18970–5. doi: 10.1073/pnas.1309699110 24191012 10.1073/pnas.1309699110 PMC3839729

[37] Wilson MS, Livermore TM, Saiardi A. Inositol pyrophosphates: between signaling and metabolism. Biochem J. 2013; 452(3):369–79. doi: 10.1042/BJ20130118 23725456 10.1042/BJ20130118

[38] Worley J, Luo X, Capaldi AP. Inositol pyrophosphates regulate cell growth and the environmental stress response by activating the HDAC Rpd3L. Cell Rep. 2013; 3(5):1476–82. doi: 10.1016/j.celrep.2013.03.043 23643537 10.1016/j.celrep.2013.03.043 PMC3672359

[39] Wu M, Chong LS, Perlman DH, Resnick AC, Fiedler D. Inositol polyphosphates intersect with signaling and metabolic networks via two distinct mechanisms. Proc Natl Acad Sci U S A. 2016; 113(44):E6757–E65. doi: 10.1073/pnas.1606853113 27791083 10.1073/pnas.1606853113 PMC5098652

[40] Wild R, Gerasimaite R, Jung JY, Truffault V, Pavlovic I, Schmidt A, et al Control of eukaryotic phosphate homeostasis by inositol polyphosphate sensor domains. Science. 2016; 352(6288):986–90. doi: 10.1126/science.aad9858 27080106 10.1126/science.aad9858

[41] Saiardi A, Bhandari R, Resnick AC, Snowman AM, Snyder SH. Phosphorylation of proteins by inositol pyrophosphates. Science. 2004; 306(5704):2101–5. doi: 10.1126/science.1103344 15604408 10.1126/science.1103344

[42] Bhandari R, Saiardi A, Ahmadibeni Y, Snowman AM, Resnick AC, Kristiansen TZ, et al Protein pyrophosphorylation by inositol pyrophosphates is a posttranslational event. Proc Natl Acad Sci U S A. 2007; 104(39):15305–10. doi: 10.1073/pnas.0707338104 17873058 10.1073/pnas.0707338104 PMC2000531

[43] Shively CA, Kweon HK, Norman KL, Mellacheruvu D, Xu T, Sheidy DT, et al Large-Scale Analysis of Kinase Signaling in Yeast Pseudohyphal Development Identifies Regulation of Ribonucleoprotein Granules. PLoS Genet. 2015; 11(10):e1005564 doi: 10.1371/journal.pgen.1005564 26447709 10.1371/journal.pgen.1005564 PMC4598065

[44] Ansari K, Martin S, Farkasovsky M, Ehbrecht IM, Kuntzel H. Phospholipase C binds to the receptor-like GPR1 protein and controls pseudohyphal differentiation in *Saccharomyces cerevisiae*. J Biol Chem. 1999; 274(42):30052–8. 10514491 10.1074/jbc.274.42.30052

[45] Azevedo C, Saiardi A. Extraction and analysis of soluble inositol polyphosphates from yeast. Nat Protoc. 2006; 1(5):2416–22. doi: 10.1038/nprot.2006.337 17406485 10.1038/nprot.2006.337

[46] Kuchin S, Vyas VK, Carlson M. Snf1 protein kinase and the repressors Nrg1 and Nrg2 regulate FLO11, haploid invasive growth, and diploid pseudohyphal differentiation. Mol Cell Biol. 2002; 22(12):3994–4000. doi: 10.1128/MCB.22.12.3994-4000.2002 12024013 10.1128/MCB.22.12.3994-4000.2002 PMC133850

[47] Bao MZ, Schwartz MA, Cantin GT, Yates JR, Madhani H. Pheromone-Dependent Destruction of the Tec1 Transcription Factor is Required for MAP Kinase Signaling Specificity in Yeast. Cell. 2004; 119:991–1000. doi: 10.1016/j.cell.2004.11.052 15620357 10.1016/j.cell.2004.11.052

[48] Bang S, Kim S, Dailey MJ, Chen Y, Moran TH, Snyder SH, et al AMP-activated protein kinase is physiologically regulated by inositol polyphosphate multikinase. Proc Natl Acad Sci U S A. 2012; 109(2):616–20. doi: 10.1073/pnas.1119751109 22203993 10.1073/pnas.1119751109 PMC3258619

[49] Song Q, Johnson C, Wilson TE, Kumar A. Pooled segregant sequencing reveals genetic determinants of yeast pseudohyphal growth. PLoS Genet. 2014; 10(8):e1004570 doi: 10.1371/journal.pgen.1004570 25144783 10.1371/journal.pgen.1004570 PMC4140661

[50] Johnson C, Kweon HK, Sheidy D, Shively CA, Mellacheruvu D, Nesvizhskii AI, et al The yeast sks1p kinase signaling network regulates pseudohyphal growth and glucose response. PLoS Genetics. 2014; 10(3):e1004183 doi: 10.1371/journal.pgen.1004183 24603354 10.1371/journal.pgen.1004183 PMC3945295

[51] Ross-Macdonald P, Coelho PS, Roemer T, Agarwal S, Kumar A, Jansen R, et al Large-scale analysis of the yeast genome by transposon tagging and gene disruption. Nature. 1999; 402(6760):413–8. doi: 10.1038/46558 10586881 10.1038/46558

[52] Mortimer RK, Johnston JR. Genealogy of principal strains of the yeast genetic stock center. Genetics. 1986; 113(1):35–43. 3519363 10.1093/genetics/113.1.35 PMC1202798

[53] Guthrie C, Fink G. Guide to Yeast Genetics and Molecular Biology. San Diego, CA: Academic Press; 1991.

[54] Ma J, Jin R, Jia X, Dobry CJ, Wang L, Reggiori F, et al An interrelationship between autophagy and filamentous growth in budding yeast. Genetics. 2007; 177(1):205–14. doi: 10.1534/genetics.107.076596 17890363 10.1534/genetics.107.076596 PMC2013727

[55] Gietz RD, Schiestl RH. High-efficiency yeast transformation using the LiAc/SS carrier DNA/PEG method. Nat Protoc. 2007; 2(1):31–4. doi: 10.1038/nprot.2007.13 17401334 10.1038/nprot.2007.13

[56] Lee KM, DaSilva NA. Evaluation of the Saccharomyces cerevisiae ADH2 promoter for protein synthesis. Yeast. 2005; 22:431–40. doi: 10.1002/yea.1221 15849781 10.1002/yea.1221

[57] Longtine MS, McKenzie III, A, Demarini DJ, Shah NG, Wach A, Brachat A, et al Additional Modules for Versatile and Economical PCR-based Gene Deletion and Modification in *Saccharomyces cerevisiae*. Yeast. 1998; 14:953–61. doi: 10.1002/(SICI)1097-0061(199807)14:10<953::AID-YEA293>3.0.CO;2-U 9717241 10.1002/(SICI)1097-0061(199807)14:10<953::AID-YEA293>3.0.CO;2-U

[58] Bharucha N, Ma J, Dobry CJ, Lawson SK, Yang Z, Kumar A. Analysis of the Yeast Kinome Reveals a Network of Regulated Protein Localization During Filamentous Growth. Mol Biol Cell. 2008; 19:2708–17. doi: 10.1091/mbc.E07-11-1199 18417610 10.1091/mbc.E07-11-1199 PMC2441683

[59] Aslanidis C, de Jong PJ. Ligation-independent cloning of PCR products (LIC-PCR). Nucleic Acids Res. 1990; 18(20):6069–74. 2235490 10.1093/nar/18.20.6069 PMC332407

[60] Gelperin D, White MA, Wilkinson ML, Kon Y, Kung LA, Wise KJ, et al Biochemical and genetic analysis of the yeast proteome with a movable ORF collection. Genes Dev. 2005; 19:2816–26. doi: 10.1101/gad.1362105 16322557 10.1101/gad.1362105 PMC1315389

[61] Zheng L, Baumann U, Reymond JL. An efficient one-step site-directed and site-saturation mutagenesis protocol. Nucleic Acids Res. 2004; 32(14):e115 doi: 10.1093/nar/gnh110 15304544 10.1093/nar/gnh110 PMC514394

[62] Ma J, Dobry CJ, Krysan DJ, Kumar A. Unconventional genomic architecture in the budding yeast *Saccharomyces cerevisiae* masks the nested antisense gene NAG1. Eukaryot Cell. 2008; 7(8):1289–98. doi: 10.1128/EC.00053-08 18310357 10.1128/EC.00053-08 PMC2519765

[63] Xu T, Shively CA, Jin R, Eckwahl MJ, Dobry CJ, Song Q, et al A profile of differentially abundant proteins at the yeast cell periphery during pseudohyphal growth. J Biol Chem. 2010; 285(20):15476–88. doi: 10.1074/jbc.M110.114926 20228058 10.1074/jbc.M110.114926 PMC2865295

