# Supplementary figures and images for: Inositol polyphosphates regulate and predict yeast pseudohyphal growth phenotypes
Source: PLoS Genet. 2018 Jun 25;14(6):e1007493. doi: 10.1371/journal.pgen.1007493 (PMC6034902; doi:10.1371/journal.pgen.1007493)

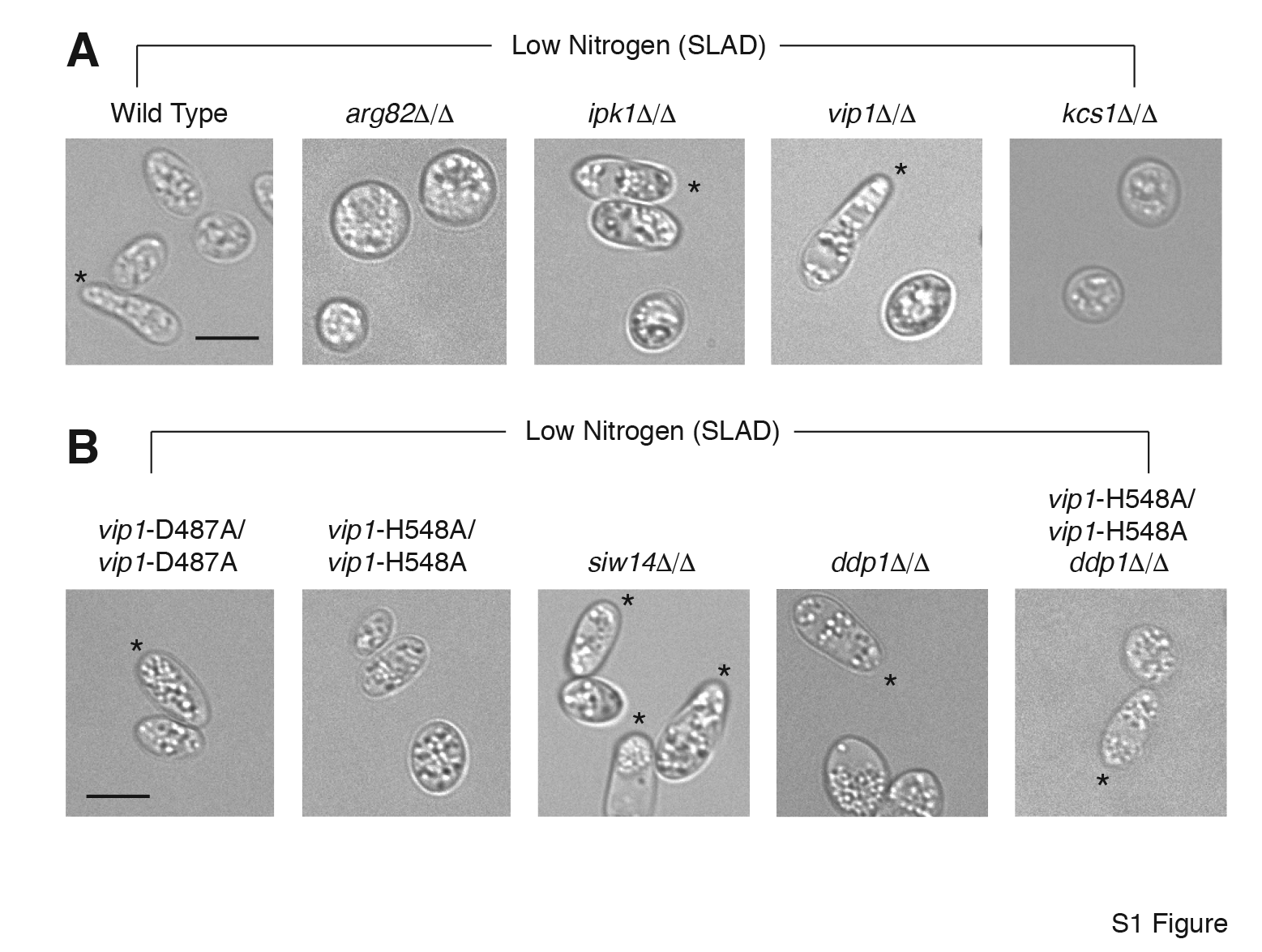

Supplement: S1 Fig — (A) The indicated strains deleted for genes encoding IP kinases were grown in low-nitrogen media along with the wild-type Σ1278b parent strain. Cells were scraped from a colony after growth in low-nitrogen SLAD medium, and the cells were suspended in solution prior to DIC imaging. Cells with length-to-width ratios greater than 2.0 are highlighted with asterisks. Scale bar, 5 μm. (B) Cell morphology of indicated strains mutated for genes encoding IP phosphatases. Cells were grown and imaged as above. Scale bar, 5 μm. (TIF) [file pgen.1007493.s001.tif]

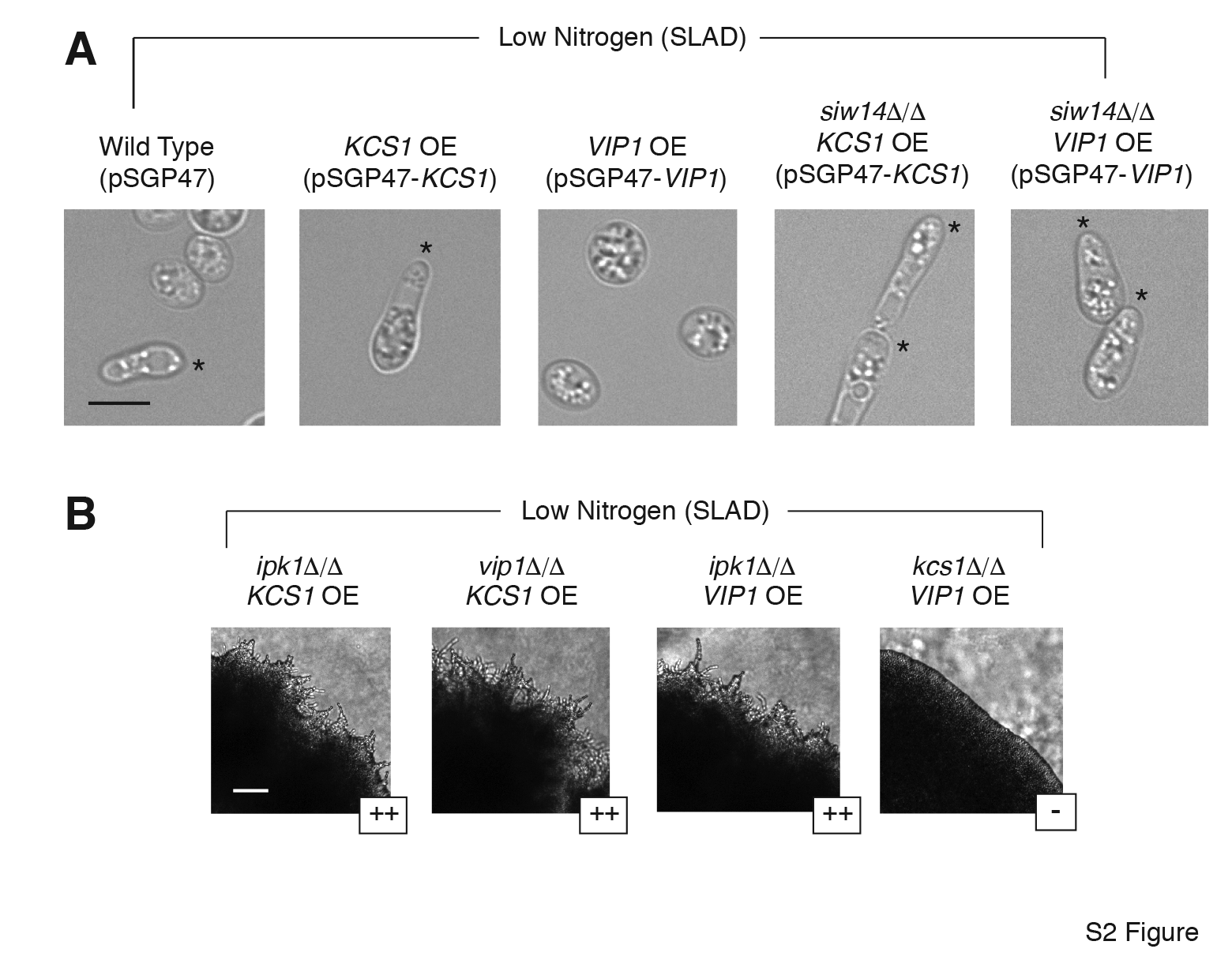

Supplement: S2 Fig — (A) Cells of the indicated strains were scraped from colonies grown on low-nitrogen SLAD medium and imaged by DIC. Cells with length-to-width ratios greater than 2.0 are highlighted with asterisks. Scale bar, 5 μm. (B) Surface-spread filamentation phenotypes of indicated mutant strains. “++” represents hyperfilamentous growth, and “-” indicates an absence of pseudohyphal filamentation. Scale bar, 500 μm. (TIF) [file pgen.1007493.s002.tif]

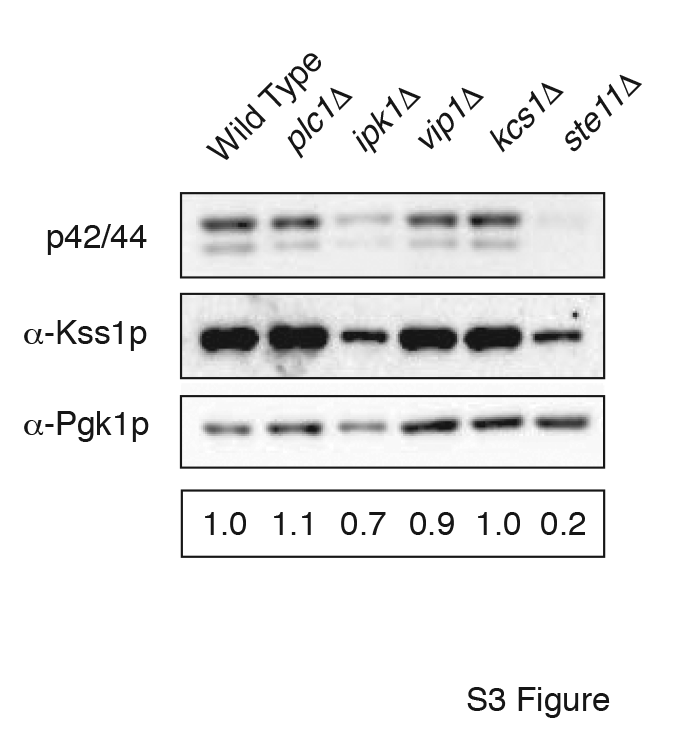

Supplement: S3 Fig — Cells were grown overnight in YPD and 30°C. Cells were sub-cultured into either YPD or YP-Gal medium and grown for 6 hours. Protein extracts were made by TCA precipitation and separated by 10% SDS-PAGE. Proteins were transferred to nitrocellulose membrane and blotted with either p44/42 antibody (Cell Signaling Technology, Danvers, MA; #4370) or Kss1 antibody (Santa Cruz Biotechnology, Santa Cruz, CA; #6775) or Pgk1 antibody (Life Technologies; Camarillo, CA; #459250) as indicated. For secondary antibodies (goat anti-mouse IgG–HRP, Bio-Rad Laboratories, Hercules, CA; #170–6516; goat anti-rabbit IgG-HRP, Jackson ImmunoResearch Laboratories, Inc., West Grove, PA; #111-035-144) were used. Membranes were developed using Western Bright kit from Advansta Inc. (Menlo Park, CA; LPS #K-12045-D20) and imaged using Imagelab software (Biorad Inc.). The ste11Δ mutant is a control for diminished Kss1p phosphorylation. Levels of phosphorylated Kss1p relative to wild-type were estimated by densitometry with normalization to the Pgk1p loading control. (TIF) [file pgen.1007493.s003.tif]

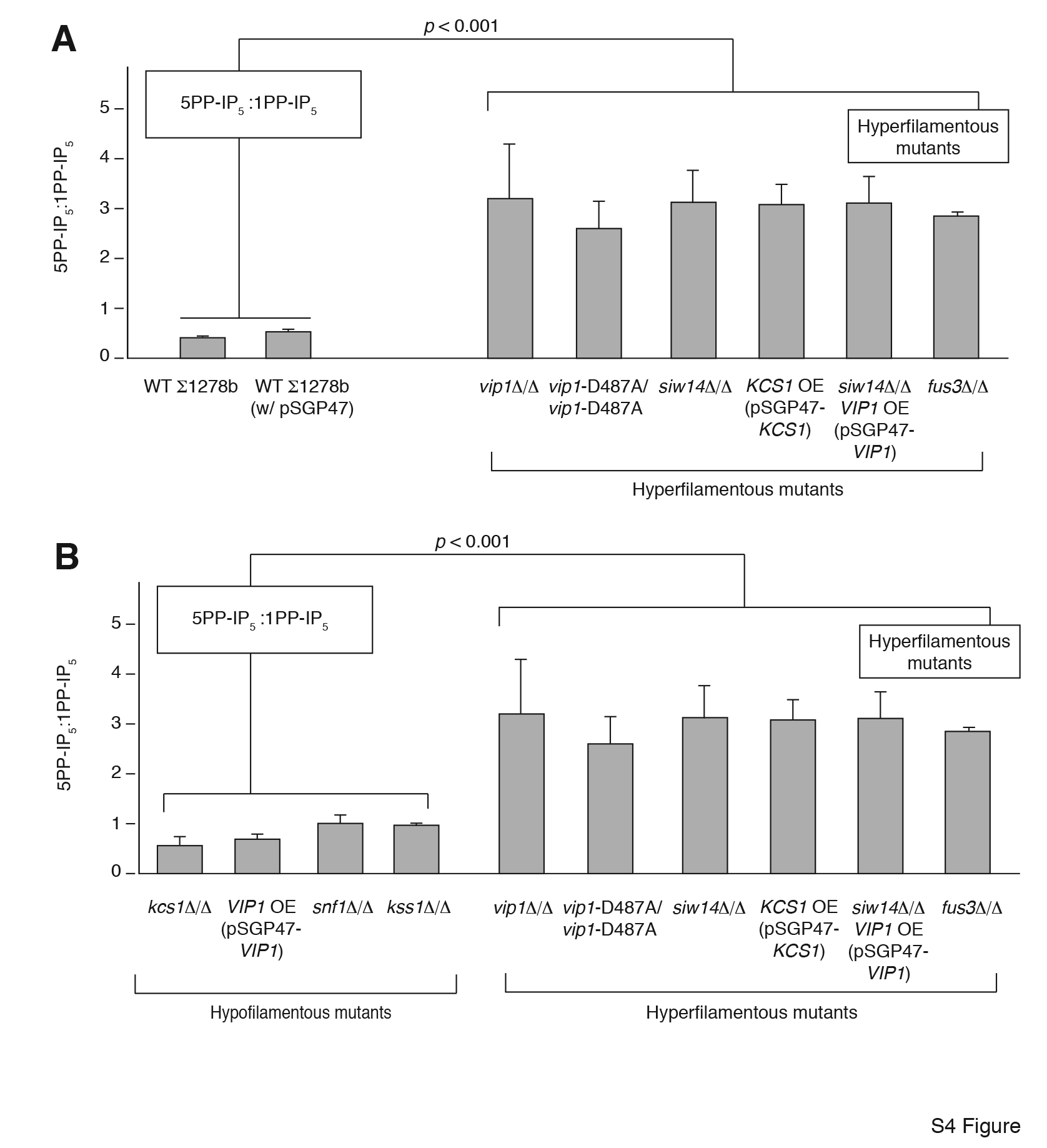

Supplement: S4 Fig — (A) By independent samples T-test, the difference in ratios of the InsP7 isoforms between hyperfilamentous mutants analyzed in this study (vip1Δ/Δ, vip1-D487A/D487A, siw14Δ/Δ, KCS1 overexpression, siw14Δ/Δ VIP1 overexpression double mutants, and fus3Δ/Δ strains) and wild-type control strains is statistically significant (p<0.001). (B) Hyperfilamentous mutants indicated above also exhibit a statistically significant difference (p<0.001) in 5PP-InsP5:1PP-InsP5 ratios from hypofilamentous mutants (kcs1Δ/Δ, VIP1 overexpression, snf1Δ/Δ, and kss1Δ/Δ mutants) analyzed in this study. (TIF) [file pgen.1007493.s004.tif]
