# Supplementary material for: Inositol polyphosphates regulate and predict yeast pseudohyphal growth phenotypes
Source: PLoS Genet. 2018 Jun 25;14(6):e1007493. doi: 10.1371/journal.pgen.1007493 (PMC6034902; doi:10.1371/journal.pgen.1007493)
Supplement: S1 Table — (RTF) [file pgen.1007493.s005.rtf]

S1 Table.  The pseudohyphal growth kinases Snf1p and Kss1p are required for wild-type phosphorylation of IP kinases
Protein	Identified Phosphosite(s)	Protein Domain	KD/WT (normalized)	Kinase-defective mutant	
Arg82p	S97	N/A	2.7	snf1-K84R	
Vip1p	S821,824	His Phosphatase Family	0.22	snf1-K84R	
Vip1p	S77	N/A	0.22	kss1-K42R	
Vip1p	T82	N/A	0.24	kss1-K42R	
Vip1p	S77	N/A	0.11	ste7-K220R	
Kcs1p	S537	His Phosphatase Family	0.23	kss1-K42R	
Kcs1p	S646	His Phosphatase Family	0.24	snf1-K84R	
The indicated kinase-defective mutants were analyzed by SILAC-based mass spectrometry to identify differentially phosphorylated proteins extracted under conditions inducing pseudohyphal growth. Phosphorylated residues on a single phosphopeptide are separated by commas. Phosphorylation sites were localized with p>0.75. The ratio of phosphopeptide in the kinase-defective mutant relative to wild type and normalized to protein level is shown. All results exhibit a statistical significance of <0.05.
