# Supplementary material for: Inositol polyphosphates regulate and predict yeast pseudohyphal growth phenotypes
Source: PLoS Genet. 2018 Jun 25;14(6):e1007493. doi: 10.1371/journal.pgen.1007493 (PMC6034902; doi:10.1371/journal.pgen.1007493)
Supplement: S2 Table — (RTF) [file pgen.1007493.s006.rtf]

S2 Table.  FLO11 mRNA levels in InsP phosphatase mutants
Straina	FLO11 mRNA levels : WTb	Pseudohyphal growth phenotypec	
Wild type	1.00 ± 0.09	WT (+) / 1.00 ± 0.04	
vip1Δ/Δ	3.08 ± 2.00	 ++ / 1.62 ± 0.04	
vip1-D487A/D487A	9.15 ± 2.94	++ / 1.67  0.11	
vip1-H548A/H548A	2.32 ± 1.03	+ / 0.96  0.11	
ddp1Δ/Δ	1.37 ±0.27	+ / 0.93  0.09	
siw14Δ/Δ	2.14 ± 0.53	++ / 2.36  0.45	
siw14Δ/Δ KCS1 OE	5.62 ± 1.77	++ / 2.08  0.09	
siw14Δ/Δ VIP1 OE	5.93 ± 0.53	++ / 1.69  0.18	
aAll indicated strains are diploid and are derived from the 1278b genetic background.
bFLO11 mRNA levels are presented for the mutants relative to levels measured in the wild-type strain under conditions of low nitrogen. Data are presented as mean with standard deviation indicated.
cPseudohyphal growth is presented as the ratio of the circumference of a colony of the indicated mutant strain to the circumference of the wild-type strain colony under conditions of low nitrogen.
 
