# Supplementary material for: Inositol polyphosphates regulate and predict yeast pseudohyphal growth phenotypes
Source: PLoS Genet. 2018 Jun 25;14(6):e1007493. doi: 10.1371/journal.pgen.1007493 (PMC6034902; doi:10.1371/journal.pgen.1007493)
Supplement: S3 Table — (RTF) [file pgen.1007493.s007.rtf]

S3 Table.  List of strains used in this study

Strain	Genotype	Source	
Y825	ura3-52 leu2Δ0 MATa 	M. Snyder (Stanford, CA)	
HLY337	ura3-52 trp1-1 MATα 	G. Fink (MIT, MA)	
Y825xHLY337	ura3-52/ura3-52 leu2Δ0 trp1-1 MATa/α		
BY4743	MATa/α his3Δ1/his3Δ1 leu2Δ0/leu2Δ0 LYS2/lys2Δ0 met15Δ0/MET15 ura3Δ0/ura3Δ0	This study	
yKN12	arg82Δ::KanMX6 ura3-52 leu2Δ0 MATa	This study	
yKN13	arg82Δ::KanMX6 ura3-52 trp1-1 MATα	This study	
yKN14	arg82Δ::KanMX6/arg82Δ::KanMX6 ura3-52/ura3-52 leu2Δ0 trp1-1 MATa/α	This study	
yKN15	ipk1Δ::KanMX6 ura3-52 leu2Δ0 MATa	This study	
yKN16	ipk1Δ::KanMX6 ura3-52 trp1-1 MATα	This study	
yKN17	ipk1Δ::KanMX6/ ipk1Δ::KanMX6 ura3-52/ura3-52 leu2Δ0 trp1-1 MATa/α	This study	
yKN18	vip1Δ::KanMX6 ura3-52 leu2Δ0 MATa	This study	
yKN19	vip1Δ::KanMX6 ura3-52 trp1-1 MATα	This study	
yKN20	vip1Δ::KanMX6/vip1Δ::KanMX6 ura3-52/ura3-52 leu2Δ0 trp1-1 MATa/α	This study	
yKN21	kcs1Δ::KanMX6 ura3-52 leu2Δ0 MATa	This study	
yKN22	kcs1Δ::KanMX6 ura3-52 trp1-1 MATα	This study	
yKN23	kcs1Δ::KanMX6/kcs1Δ::KanMX6 ura3-52/ura3-52 leu2Δ0 trp1-1 MATa/α	This study	
yKN24	ddp1Δ::KanMX6 ura3-52 leu2Δ0 MATa	This study	
yKN25	ddp1Δ::KanMX6 ura3-52 trp1-1 MATα	This study	
yKN26	ddp1Δ::KanMX6/ddp1Δ::KanMX6 ura3-52/ura3-52 leu2Δ0 trp1-1 MATa/α	This study	
yKN27	siw14Δ::KanMX6 ura3-52 leu2Δ0 MATa	This study	
yKN28	siw14Δ::KanMX6 ura3-52 trp1-1 MATα	This study	
yKN29	siw14Δ::KanMX6/siw14Δ::KanMX6 ura3-52/ura3-52 leu2Δ0 trp1-1 MATa/α	This study	
yKN30	ura3-52/ura3-52 leu2Δ0 trp1-1 MATa/α + pSGP47-KCS1	This study	
yKN31	ura3-52/ura3-52 leu2Δ0 trp1-1 MATa/α + pSGP47-VIP1	This study	
yKN32	fus3Δ::HphMX4/fus3Δ::KanMX6 ura3-52/ura3-52 leu2Δ0 trp1-1 MATa/α	This study	
yKN33	kss1Δ::HphMX4/kss1Δ::KanMX6 ura3-52/ura3-52 leu2Δ0 trp1-1 MATa/α	This study	
yKN34	snf1Δ::KanMX6 vip1Δ::HphMX4 ura3-52 leu2Δ0 MATa	This study	
yKN35	snf1Δ::KanMX6 vip1Δ::HphMX4 ura3-52 trp1-1 MATα	This study	
yKN36	snf1Δ::KanMX6/ snf1Δ::KanMX6 vip1Δ::HphMX4/ vip1Δ::HphMX4 ura3-52/ura3-52 leu2Δ0 trp1-1 MATa/α	This study	
yKN37	snf1Δ::KanMX6/snf1Δ::KanMX6 ura3-52/ura3-52 leu2Δ0 trp1-1 MATa/α + pSGP47-VIP1	This study	
yKN38	snf1Δ::KanMX6/snf1Δ::KanMX6 ura3-52/ura3-52 leu2Δ0 trp1-1 MATa/α + pSGP47-KCS1	This study	
yKN39	ipk1Δ::KanMX6/ ipk1Δ::KanMX6 ura3-52/ura3-52 leu2Δ0 trp1-1 MATa/α + pSGP47-KCS1	This study	
yKN40	ipk1Δ::KanMX6/ ipk1Δ::KanMX6 ura3-52/ura3-52 leu2Δ0 trp1-1 MATa/α + pSGP47-VIP1	This study	
yKN41	vip1Δ::KanMX6/vip1Δ::KanMX6 ura3-52/ura3-52 leu2Δ0 trp1-1 MATa/α + pSGP47-KCS1	This study	
yKN42	kcs1Δ::KanMX6/kcs1Δ::KanMX6 ura3-52/ura3-52 leu2Δ0 trp1-1 MATa/α + pSGP47-VIP1	This study	
yKN43	siw14Δ::KanMX6/siw14Δ::KanMX6 ura3-52/ura3-52 leu2Δ0 trp1-1 MATa/α + pSGP47-KCS1	This study	
yKN44	siw14Δ::KanMX6/siw14Δ::KanMX6 ura3-52/ura3-52 leu2Δ0 trp1-1 MATa/α + pSGP47-VIP1	This study	
yCS1	snf1Δ::KanMX6 ura3-52 leu2Δ0 MATa	This study	
yCS2	snf1Δ::KanMX6 ura3-52 trp1-1 MATα	This study	
yCS3	snf1Δ::KanMX6/snf1Δ::KanMX6 ura3-52/ura3-52 leu2Δ0 trp1-1 MATa/α	This study	
