# Supplementary material for: Inositol polyphosphates regulate and predict yeast pseudohyphal growth phenotypes
Source: PLoS Genet. 2018 Jun 25;14(6):e1007493. doi: 10.1371/journal.pgen.1007493 (PMC6034902; doi:10.1371/journal.pgen.1007493)
Supplement: S4 Table — (RTF) [file pgen.1007493.s008.rtf]

S4 Table.  mRNA levels of over-expressed KCS1 and VIP1 in high-copy vectors with the ADH2 promoter
Strain	Growth condition	Gene	mRNA levelsa	
Wild type	YPD (Normal N)	KCS1	0.73 ± 0.40	
KCS1 OE (pSGP47-KCS1)	SC –Ura (Normal N)	KCS1	17.3 ± 7.6	
	SLAD (Low N)	KCS1	526 ± 114	
Wild type	YPD (Normal N)	VIP1	1.40 ± 0.09	
VIP1 OE (pSGP47-VIP1)	SC –Ura (Normal N)	VIP1	37.5 ± 18.8	
	SLAD (Low N)	VIP1	587 ± 174	
aQuantified as fold-change relative to levels in WT strain grown in SLAD
