# Supplementary material for: Inositol polyphosphates regulate and predict yeast pseudohyphal growth phenotypes
Source: PLoS Genet. 2018 Jun 25;14(6):e1007493. doi: 10.1371/journal.pgen.1007493 (PMC6034902; doi:10.1371/journal.pgen.1007493)
Supplement: S5 Table — (RTF) [file pgen.1007493.s009.rtf]

S5 Table.  Invasive growth assay datasets
Yeast strain	Dataset	Invasive growth (pixel intensity post-wash/pre-wash)	
Wild type  (Σ1278b)	Replicate 1
Replicate 2
Replicate 3	0.52
0.64
0.55	
arg82Δ	Replicate 1
Replicate 2
Replicate 3	0.32
0.33
0.40	
ipk1Δ


vip1Δ


kcs1Δ


vip1-D487A


vip1-H548A	Replicate 1
Replicate 2
Replicate 3
Replicate 1
Replicate 2
Replicate 3
Replicate 1
Replicate 2
Replicate 3
Replicate 1
Replicate 2
Replicate 3
Replicate 1
Replicate 2
Replicate 3	0.76
0.65
0.81
0.81
0.87
0.90
0.26
0.30
0.27
0.87
0.63
0.68
0.58
0.53
0.53	
