# Supplementary material for: Inositol polyphosphates regulate and predict yeast pseudohyphal growth phenotypes
Source: PLoS Genet. 2018 Jun 25;14(6):e1007493. doi: 10.1371/journal.pgen.1007493 (PMC6034902; doi:10.1371/journal.pgen.1007493)
Supplement: S6 Table — (RTF) [file pgen.1007493.s010.rtf]

S6 Table.  Pseudohyphal filament circumference of independent replicate colonies
Yeast strain	Dataset	Circumference relative to WT under inducing conditions (Low N)	
Wild type  (Σ1278b)	Replicate 1
Replicate 2
Replicate 3	1.00
1.04
0.96	
arg82Δ/Δ	Replicate 1
Replicate 2
Replicate 3	0.12
0.26
0.11	
ipk1Δ/Δ


vip1Δ/Δ


kcs1Δ/Δ


vip1-D487A/vip1-D487A


vip1-H548A/vip1-H548A


siw14Δ/Δ


ddp1Δ/Δ


vip1-H548A/vip1-H548A ddp1Δ/Δ

Wild type (Σ1278b with pSGP47)


KCS1 OE (pSGP47-KCS1)


VIP1 OE (pSGP47-VIP1)


siw14Δ/Δ KCS1 OE (pSGP47-KCS1)

siw14Δ/Δ VIP1 OE (pSGP47-VIP1)

Wild type (Σ1278b with pSGP47 in media with normal N levels) 

KCS1 OE (Normal N levels)


VIP1 OE (Normal N levels)


siw14Δ/Δ (Normal N levels)


siw14Δ/Δ KCS1 OE (Normal N)


siw14Δ/Δ VIP1 OE (Normal N) 	Replicate 1
Replicate 2
Replicate 3
Replicate 1
Replicate 2
Replicate 3
Replicate 1
Replicate 2
Replicate 3
Replicate 1
Replicate 2
Replicate 3
Replicate 1
Replicate 2
Replicate 3
Replicate 1
Replicate 2
Replicate 3
Replicate 1
Replicate 2
Replicate 3
Replicate 1
Replicate 2
Replicate 3
Replicate 1
Replicate 2
Replicate 3
Replicate 1
Replicate 2
Replicate 3
Replicate 1
Replicate 2
Replicate 3
Replicate 1
Replicate 2
Replicate 3
Replicate 1
Replicate 2
Replicate 3
Replicate 1
Replicate 2
Replicate 3
Replicate 1
Replicate 2
Replicate 3
Replicate 1
Replicate 2
Replicate 3
Replicate 1
Replicate 2
Replicate 3
Replicate 1
Replicate 2
Replicate 3
Replicate 1	1.80
1.78
1.74
1.68
1.60
1.58
0.04
0.11
0.09
1.79
1.53
1.68
0.84
0.92
1.11
2.81
1.75
2.53
0.91
0.83
1.04
0.94
1.11
0.83
1.00
1.20
0.80
2.30
2.13
2.35
0.58
0.45
0.34
2.20
1.98
2.07
1.92
1.68
1.47
0.65
0.55
0.60
0.99
1.13
1.04
0.63
0.55
0.75
1.36
1.25
1.14
1.47
1.40
1.26
0.62	
	Replicate 2	0.71	
	Replicate 3	0.48	
