# Supplementary material for: Inositol polyphosphates regulate and predict yeast pseudohyphal growth phenotypes
Source: PLoS Genet. 2018 Jun 25;14(6):e1007493. doi: 10.1371/journal.pgen.1007493 (PMC6034902; doi:10.1371/journal.pgen.1007493)
Supplement: S7 Table — (RTF) [file pgen.1007493.s011.rtf]

S7 Table.  Ratio of InsP7 isoforms in independent replicates for indicated strains
Yeast strain	Growth condition	Dataset	5PP-InsP5:1PP-InsP5	
Wild type  (Σ1278b)	Minimal media Low N	Replicate 1

Replicate 2	0.44

0.49	
Wild type  (BY4743)	Minimal media Low N	Replicate 1

Replicate 2	0.78

0.70	
vip1Δ/Δ


kcs1Δ/Δ


vip1-D487A/vip1-D487A

vip1-H548A/vip1-H548A

ddp1Δ/Δ


vip1-H548A/vip1-H548A ddp1Δ/Δ

siw14Δ/Δ


Wild type (Σ1278b with pSGP47)

VIP1 OE (pSGP47-VIP1)

KCS1 OE (pSGP47-
KCS1)

siw14Δ/Δ VIP1 OE


WT (Σ1278b with pSGP47)


KCS1 OE (pSGP47-KCS1)	Minimal media Low N

Minimal media Low N

Minimal media Low N

Minimal media Low N

Minimal media Low N

Minimal media
Low N

Minimal media
Low N

Minimal media
-Ura, Low N

Minimal media, 
-Ura, Low N

Minimal media. 
–Ura, Low N

Minimal media,
-Ura, Low N

Media with normal N levels, -Ura


Media with normal N levels, -Ura	Replicate 1

Replicate 2
Replicate1

Replicate 2
Replicate 1

Replicate 2
Replicate 1

Replicate 2
Replicate 1

Replicate 2
Replicate 1

Replicate 2
Replicate 1

Replicate 2
Replicate 1

Replicate 2
Replicate 1

Replicate 2
Replicate 1

Replicate 2
Replicate 1

Replicate 2
Replicate 1

Replicate 2
Replicate 3
Replicate 1

Replicate 2	4.34

2.05
0.39

0.73
3.23

2.08
0.73

0.70
0.05

0.28
0.06

0.24
3.87

2.53
0.58

0.49
0.78

0.59
3.50

2.66
2.55

3.68
0.16

0.21
0.23
0.45

0.65	
snf1Δ/Δ	Minimal media, Low N	Replicate 1

Replicate 2	0.83

0.81	
fus3Δ/Δ	Minimal media, Low N	Replicate 1

Replicate 2	2.97

2.73	
kss1Δ/Δ	Minimal media,	Replicate 1	1.13	
	Low N	
Replicate 2	
0.81	
